# Microglia RAGE exacerbates the progression of neurodegeneration within the *SOD1*^*G93A*^ murine model of amyotrophic lateral sclerosis in a sex-dependent manner

**DOI:** 10.1186/s12974-021-02191-2

**Published:** 2021-06-15

**Authors:** Michael MacLean, Judyta Juranek, Swetha Cuddapah, Raquel López-Díez, Henry H. Ruiz, Jiyuan Hu, Laura Frye, Huilin Li, Paul F. Gugger, Ann Marie Schmidt

**Affiliations:** 1 Diabetes Research Program, Department of Medicine, New York University Grossman School of Medicine, New York, NY 10016 USA; 2 Department of Human Physiology and Pathophysiology, School of Medicine, University of Warmia and Mazury, Olsztyn, Poland; 3 Division of Biostatistics, Department of Population Health and the Department of Environmental Medicine, New York University Grossman School of Medicine, New York, NY 10016 USA

**Keywords:** ALS, Microglia, Neurodegeneration, Macrophage, Astrocytes, RAGE

## Abstract

**Background:**

Burgeoning evidence highlights seminal roles for microglia in the pathogenesis of neurodegenerative diseases including amyotrophic lateral sclerosis (ALS). The receptor for advanced glycation end products (RAGE) binds ligands relevant to ALS that accumulate in the diseased spinal cord and RAGE has been previously implicated in the progression of ALS pathology.

**Methods:**

We generated a novel mouse model to temporally delete *Ager* from microglia in the murine *SOD1*^*G93A*^ model of ALS. Microglia *Ager* deficient *SOD1*^*G93A*^ mice and controls were examined for changes in survival, motor function, gliosis, motor neuron numbers, and transcriptomic analyses of lumbar spinal cord. Furthermore, we examined bulk-RNA-sequencing transcriptomic analyses of human ALS cervical spinal cord.

**Results:**

Transcriptomic analysis of human cervical spinal cord reveals a range of *AGER* expression in ALS patients, which was negatively correlated with age at disease onset and death or tracheostomy. The degree of *AGER* expression related to differential expression of pathways involved in extracellular matrix, lipid metabolism, and intercellular communication. Microglia display increased RAGE immunoreactivity in the spinal cords of high *AGER* expressing patients and in the *SOD1*^*G93A*^ murine model of ALS vs. respective controls. We demonstrate that microglia *Ager* deletion at the age of symptomatic onset, day 90, in *SOD1*^*G93A*^ mice extends survival in male but not female mice. Critically, many of the pathways identified in human ALS patients that accompanied increased *AGER* expression were significantly ameliorated by microglia *Ager* deletion in male *SOD1*^*G93A*^ mice.

**Conclusions:**

Our results indicate that microglia RAGE disrupts communications with cell types including astrocytes and neurons, intercellular communication pathways that divert microglia from a homeostatic to an inflammatory and tissue-injurious program. In totality, microglia RAGE contributes to the progression of *SOD1*^*G93A*^ murine pathology in male mice and may be relevant in human disease.

**Supplementary Information:**

The online version contains supplementary material available at 10.1186/s12974-021-02191-2.

## Background

Amyotrophic lateral sclerosis (ALS), a progressive, fatal, neurodegenerative disease, is characterized by the inexorable death of motor neurons. Affected patients experience progressive muscle atrophy, motor function decline, and eventual paralysis. ALS rapidly progresses in most patients, resulting in an estimated survival of 2–4 years after onset of symptoms [1]. Greater than 90% of ALS patients have no known family history of the disease and hence the non-familial form of the disease is known as sporadic ALS (sALS). A smaller subset of patients (5–10%) is affected by familial ALS (fALS) [1]. Mutations in superoxide dismutase 1 (*SOD1*) account for approximately 20% of fALS patients [1]. Regardless of the underlying disease-related mutation, ALS is characterized by the accumulation of proteinaceous aggregates, which is associated with a pro-damage inflammatory and oxidative stress state involving multiple cell types. Ultimately, these processes collectively augur motor neuron death [2, 3]. Mice carrying multiple copies of a human *SOD1*^*G93A*^ transgene display hallmarks of ALS, including accumulation of proteinaceous aggregates, neuroinflammation, and progressive motor function decline with eventual paralysis and death in all *SOD1*^*G93A*^ transgenic mice [4, 5]. While ALS results in motor neuron death, several landmark studies have elucidated critical roles for non-neuronal cells during disease progression [6–11]. Altogether, these considerations illustrate that ALS pathology is driven by dysfunction across a myriad of cell types whose intercommunications drive processes that irreparably damage neurons.

Spatial transcriptomic analysis of *SOD1*^*G93A*^ mouse spinal cords suggests that microglial dysfunction is evident by post-natal day 30 (P30), with astrocyte dysfunction becoming apparent later, by P70. These findings suggest that microglial dysfunction may precede and contribute to astrocyte dysfunction, which precedes neuronal death [12]. Critically, these key findings implicating glial cells in ALS pathobiology were also observed in spatial transcriptomics analyses of human ALS spinal cord [12]. Studies in *SOD1*^*G93A*^ mice affirmed mediating roles for glial cells in impacting survival in *SOD1*^*G93A*^ mice; when the myeloid and lymphoid compartment of these mice was replaced with a wild-type (WT) bone marrow transplant within 24 h of birth, the recipient *SOD1*^*G93A*^ mice demonstrated improved survival compared to those *SOD1*^*G93A*^ mice receiving *SOD1*^*G93A*^ bone marrow [9]. Moreover, an expanding number of genes with known ALS-linked mutations mediate myeloid function, including *C9ORF72*, *TARDP*, and *OPTN* [13, 14]. Specifically, *C9ORF72* regulates phagosome and lysosome pathways [15], while *TARDP* regulates phagocytosis [16]. *OPTN* deficiency induces an inflammatory profile in microglia [17]. It has previously been shown that reducing myeloid inflammation increases lifespan of *SOD1*^*G93A*^ mice [7, 18]. In a distinct inducible murine model of ALS, microglia were required for the clearance of protein aggregates [19]. Microglia from *SOD1*^*G93A*^ mice display unique transcriptomic signatures reminiscent of both protective and inflammatory macrophages [20]. Altogether, these findings suggest that microglia may exert opposing roles in ALS, mediating stage-dependent alterations in protective vs. damage-provoking functions. In fact, accruing evidence suggests that microglia may undergo step-wise activation toward a dysfunctional inflammatory disease-associated microglia (DAM) phenotype, at least in the context of Alzheimer’s disease (AD) [21, 22]. Triggering receptor expressed on myeloid cells 2 (TREM2) has been implicated in the step-wise activation of microglia toward this DAM state; however, it is established that additional factors synergize or act independently of TREM2 in this “activation pathway” and that TREM2 plays complex pro- and anti-inflammatory effects in immune cells [23]. The identity of such additional factors is thus essential to discover, as they may unveil new therapeutic targets for neurodegenerative disorders.

The receptor for advanced glycation end products (RAGE) is an immunoglobulin (Ig)-type transmembrane receptor expressed by many cell types and is upregulated in patient and murine ALS tissues [24–26]. RAGE binds a diverse set of ligands, particularly those noted as damage associated molecular patterns (DAMPs), including several upregulated in ALS patient and *SOD1*^*G93A*^ mouse spinal cord, such as S100 calcium binding protein B, carboxymethyllysine (CML)-advanced glycation end product (AGE), and high-mobility group box 1 (HMGB1) [25, 27–29]. Spatial transcriptomic analyses recently identified an enrichment of the “AGE-RAGE pathway in diabetic complications” Kyoto Encyclopedia of Genes and Genomes (KEGG) pathway in both murine and human ALS spinal cord within glia-related modules of gene expression [12].

While the discrete role of microglia RAGE in the context of ALS has never been studied, RAGE activation on myeloid cells, including microglia, stimulates NF-κB activity, thereby promoting pro-inflammatory cytokine expression and generation of reactive oxygen species [30–34]. Ligand engagement with RAGE induces a conformational change in its cytoplasmic tail that signals, at least in part, through diaphanous related formin 1 (DIAPH1) [35, 36]. Microglia have been shown to induce motor neuron death and drive pathology in *SOD1*^*G93A*^ mice via activation of NF-κB [7]. We hypothesize that microglia RAGE contributes to oxidative stress and exacerbation of pro-inflammatory gene expression, processes which divert microglia from their homeostatic and protective functions, thereby redirecting them to a phenotype that contributes to neurodegeneration.

In the current study, analysis of human ALS patient RNA-sequencing data uncovered for the first time that a spectrum of expression of *AGER*, the gene encoding RAGE, was evident in ALS cervical spinal cord tissue, and that this was related to alterations in pathways involving lipid metabolism, cellular microenvironment, and intercellular communication. Furthermore, patients with high *AGER* expression displayed increased levels of RAGE in microglia in the cervical spinal cord. In a murine model, we demonstrate a significant survival benefit and improvement in motor function performance in male but not female microglia *Ager*-deficient *SOD1*^*G93A*^ *Ager*^fl/fl^ *Cx3cr1*^Cre/+^ mice (microglia *Ager* deletion) relative to *SOD1*^*G93A*^ *Ager*^+/+^ *Cx3cr1*^Cre/+^ mice (Cre-controls; microglia express *Ager*) subjected to deletion of microglia *Ager* at 3 months of age. Remarkably, deletion of microglia *Ager* from *SOD1*^*G93A*^ mice modulated the lumbar spinal cord transcriptome in a manner such that a number of pathways related to increased *AGER* expression in human ALS patients were significantly ameliorated. Altogether, these data suggest that RAGE may contribute to the pathological re-programming of innate microglia functions in the ALS spinal cord.

## Materials and methods

### Animals

All experiments were performed on C57BL/6J mice intercrossed with the following strains: B6.129P2(Cg)-CX3CR1^tm2.1(cre/ERT2)Litt/WganJ^ (JAX Stock No: 021160), *Ager*^*Flox/Flox*^ mice (C56BL6J background), and B6.Cg-Tg(SOD1G93A)1Gur/J (JAX Stock No:004435) [4, 37–39]. Note that all mice employed in this study were extensively backcrossed into or generated directly in C57BL/6J background. In all mice, copy number was analyzed and any mice with alterations were not enrolled in the study [40]. Three mice over the course of the study were censored due to non-ALS deaths. All mice were maintained under pathogen-free conditions. Unless otherwise noted, all mice were housed in a temperature (19–23 °C) and humidity (30–70% relative humidity)-regulated environment with 12-h light/dark cycle, lights on/off at 6:30, and received standard chow food pellets (Lab Diets, Cat: 5053) and water ad libitum. Tamoxifen (TAM) was used to delete *Ager* from *Cx3cr1*-expressing cells at 90 days of age. All animals, irrespective of genotype, were administered 0.2 μg of TAM (Sigma, Cat: T5648) dissolved in corn oil (Sigma-Aldrich, Cat: C8267) once daily every day for a total of five intraperitoneal injections except for the female control mice used in validation of *Ager* deletion, which received five intraperitoneal injections administered every other day. Onset of disease was identified by the age at which the animal reached maximum weight. The humane endpoint was determined by 20% weight loss from maximum weight, or the inability of the animal to right itself within 15 s of being placed on its side. When a mouse reached the humane endpoint, by either criterion, it was immediately sacrificed and date of death was recorded for survival analyses. Mice were weighed 2–3 times weekly, same time of day, with calibrated balances.

### Motor function

Measurement of motor function was performed using the Hanging Wire test as detailed previously [41]. Briefly, mice were placed on a cage wire lid, inverted 50 cm above padding and observed for up to 1 min. The amount of time until the mouse fell to the padding was recorded. All mice were trained to the procedure for 1 week prior to data acquisition (8 weeks of age). All tests were performed twice-weekly until the mice were no longer able to perform the test. In all cases, the operator was naïve to experimental code. Triplicate measurements were taken and mean value was recorded for each session and weekly means were calculated. Animals were given 30-s breaks between test replicates.

### Immunohistochemistry

#### Murine tissue staining

Mice were anesthetized with Ketamine and Xylazine and then underwent rapid cardiac perfusion with PBS, followed by 4% paraformaldehyde (PFA). The spinal column was rapidly removed, and the lumbar spinal cord was retrieved. Lumbar spinal cords were washed in PBS and then drop-fixed in 4% PFA for an additional 1 h at 4 °C. Gastrocnemius tissue was collected and drop-fixed 16 h in 1% PFA at 4 °C. After fixation, tissue was incubated with 15% sucrose in PBS for 24 h followed by incubation with 30% sucrose in PBS for an additional 24 h. Cyroprotected tissue was frozen in optimal cutting temperature (OCT) compound (FisherScientific, Cat: 23-730-571) and kept at − 80 °C until sectioning. Furthermore, 8-μm-thick serial sections were collected on Superfrost PLUS slides (FisherScientific, Cat: 22-037-246) using a Microm cryostat (ThermoFisher, Model: HM550). Sections were washed 3× with PBS for 5 min and then permeabilized with 0.2% Triton-X 100 in PBS for 10 min and washed 3× with PBS. Blocking was conducted with Serum-Free blocking buffer (Dako, Cat: X090930-2) for 1 h at RT. All primary antibodies were diluted in Antibody Diluent (Dako, Cat: S3022) and applied overnight at 4 °C. Subsequently, slides were washed 3× with PBS, then incubated with secondary antibodies diluted in Antibody Diluent for 1 h at RT. For slides containing DAPI, slides were washed and placed in a 1 μg/mL DAPI (Invitrogen, Cat: D1306) solution for 5 min at RT and washed 3× with PBS before mounting with fluorescent mounting media (Dako, Cat: S302380-2). Primary antibodies used: 1 μg/mL Mouse anti-RAGE (Millipore, Cat: MAB5328), 0.5 μg/mL Rat anti-CD11B (M1/70) (Invitrogen, Cat:14-0112-82), 0.25 μg/mL Rat anti-GFAP (2.2B10) (Invitrogen, Cat: 13-0300), 5 μg/mL Rat anti-CLEC7A (Invivogen, Cat: mabg-mdect), 1 μg/mL Mouse anti-AMIGO2 (G-7) (SantaCruz, Cat: sc-373699), 5 μg/mL Mouse anti-NeuN (Millipore, Cat: MAB377), 5 μg/mL Rat anti-CD68 (Abcam, Cat:ab53444), 5 μg/mL Rat anti-F4/80 (Abcam, Cat: ab6640), and 3 μg/mL Chicken anti-MAP2 (Abcam, Cat:ab5392). Secondary antibodies utilized: Donkey anti-Rat Alexa Fluor 488 (Invitrogen, Cat: A-21208), Donkey anti-Mouse Alexa Fluor 546 (Invitrogen, Cat: A10036), Donkey anti-Rat Alexa Fluor 594 (Invitrogen, Cat: A-21209), Donkey anti-Mouse Alexa Fluor 488 (Invitrogen, Cat: A21203), and Donkey anti-Chicken Alexa Fluor 647 (Jackson ImmunoResearch, Cat: 703-605-155). All secondary antibodies were used at 1 μg/mL. All experiments included negative controls by omission of primary antibody.

#### Human tissue staining

De-identified paraffin-embedded tissue sections from sporadic ALS patient and control cervical spinal cord tissue were provided by the Target ALS Multicenter Postmortem Tissue Core (www.targetals.org). Then, 3 × 5 min washes with Clear-Rite 3 (ThermoFisher, Cat: 6901TS) were used to de-paraffinize the sections. Slides were rehydrated in a series of 5 min EtOH washes (2 × 100%, 1 × 90%, 1 × 70%) and then washed 3× with ddH20. Antigen retrieval was performed by steaming in Epitope Retrieval Solution (IHC World, Cat: IW-1100) for 1 h then washed 3× with PBS. Sections were permeabilized for 10 min with 0.4% Triton X100 in PBS then washed 3× with PBS. Autofluorescence was reduced by incubating with 1× TrueBlack in 70% ethanol (Biotium, Cat: 23007) for 30 s, then washed 3× with large volumes of PBS. Blocking was conducted with 5% Donkey Serum (SigmaAldrich, Cat: D9663) in PBS overnight at 4 °C. All primary antibodies were diluted in 2.5% Donkey Serum in PBS and applied for 24 h at 4 °C. Subsequently, slides were washed 3× with PBS, then incubated in secondary antibodies diluted in 2.5% Donkey Serum in PBS for 1 h at RT. Slides were then washed 3× with PBS before mounting with fluorescent mounting media. Primary antibodies utilized: 5 μg/mL Rabbit anti-IBA1 (Wako, Cat: 013-27691), 4 μg/mL Goat anti-RAGE (R&D, Cat: AF1179), 5 μg/mL Mouse anti-GFAP (BD Biosciences, Cat: 556330), and 5 μg/mL Mouse anti-NeuN (Millipore, Cat: MAB377). Secondary antibodies utilized: Donkey anti-rabbit Alexa Fluor 488 (Invitrogen, Cat: A21206), Donkey anti-mouse Alexa Fluor 488 (Invitrogen, Cat: A21203), Donkey anti-Goat Alexa Fluor 594 (Invitrogen, Cat: A32758), and Donkey anti-Goat Alexa Fluor 647 (Invitrogen, Cat: A32849). All secondary antibodies were used at 1 μg/mL. As above, all experiments included negative controls with omission of primary antibodies.

### Imaging and quantification

Multicolor wide-field images were taken on a Leica 5500B microscope at × 20 or × 40 magnification, as indicated. All microscope settings were kept identical for each experiment. For mouse tissue imaging: 2–4 images/tissue slice and 3–4 tissue slices/sample were collected for analysis. For human tissue imaging: 2–4 images/tissue slice and 3 tissues slices/patient were collected for analysis, four regions of interest per image,150 × 150 μm in size, were selected as to exclude auto-fluorescent blood vessels for each image before further analysis. All analyses were performed with the Fiji distribution of ImageJ (NIH) [42].

#### Cell number analyses

Cell numbers that displayed overlap of the designated antibodies with DAPI were manually counted per image and averaged per mouse. All analysis was completed by a naïve experimenter blinded to all experimental codes.

#### Cell area analysis

To quantify the positive area of each stain in end-stage murine and human tissues, the images underwent background removal with the rolling ball radius set to 50 within ImageJ. Images were then subjected to automated thresholding with the optimal thresholding algorithm of each signal being selected by a naïve experimenter. The following thresholding algorithms were utilized: Li for: CD11B in Wild-Type vs *SOD1*^*G93A*^ mice; Moments used for: RAGE, c-Type lectin domain containing 7a protein (or dectin-1) (CLEC7A), glial fibrillary acidic protein (GFAP), microtubule-associated protein 2 (MAP2), and adhesion molecule with Ig like domain 2 (AMIGO2); Triangle used for: CD11B, CLEC7A, on 120 day old tissue, and F4/80, CD68, and neuronal nuclei antigen (NeuN); and Otsu for: human ionized calcium-binding adapter molecule 1 (IBA1), and human GFAP [43–45]. Calculation of positive area was calculated per μm^2^ and the average of 4–8 images per sample was used for statistical analysis.

### Cell culture

BV2 mouse microglia-like cells were obtained as a generous gift from Dr. Colin K. Combs (University of Nebraska). BV2 cells were grown in growth medium (Dulbecco’s modified Eagle’s medium (DMEM) (Thermofisher, Cat:11885-084) containing 10% (v/v) heat-inactivated fetal bovine serum (FBS) (Corning, Cat:35-010-CV)) with 100 units/mL penicillin and 100 μg/mL streptomycin (Thermofisher, Cat:10378016). The cells were incubated at 37 °C under 5% CO_2_ and 95% relative humidity.

### Lentivirus transfection

BV2 cells were grown to 50% confluency in normal growth medium. Growth medium was replaced with fresh growth medium supplemented with 5 μg/mL polybrene (Sigma, Cat:TR-1003) and 1.5 × 10^6^ lentiviral particles of either sh*Ager* (Sigma, Cat:TRCN0000071745) or non-targeting shRNA control (Sigma, Cat: SHC002H) lentivirus for 8 h. When cells reached 90% confluency, shRNA expressing cells were selected using growth medium with 4 μg/mL puromycin (Thermofisher, Cat: A1113803) and were maintained under puromycin selection for at least three passages before use.

### Treatment of BV2 cells

BV2 cells were plated in a 6-well plate at 3 × 10^5^ cells per well and cultured in growth medium containing 1% FBS overnight. Cells were treated with 300 μg/mL RAGE ligand CML-AGE or human serum albumin control for 24 h before lysing in Qiazol reagent (QIAGEN Inc, Cat: 79306) and frozen until RNA was isolated [46]. For RAGE inhibitor studies, cells were first pre-treated with either 10 μM RAGE inhibitor (a C11 analog) or 0.1% v/v dimethyl sulfoxide (DMSO) in 1% FBS containing growth medium for 2 h before treatment [32].

### CD11B^+^ cell isolation via autoMACS

Mice were deeply anesthetized with Ketamine and Xylazine and then cardiac-perfused with 50 mL of ice-cold PBS. Subsequently, mice were decapitated and the brain was removed and collected into ice-cold tubes containing PBS. Brain tissue was dissociated using the Adult Brain Tissue Dissociation Kit (Miltenyi, Cat: 130-107-677) as per manufacturer’s guidelines with the gentleMACS dissociator (Miltenyi, Cat:130-093-235) and strained with 70 μm filters (Miltenyi, Cat: 130-098-462). Homogenates underwent debris removal as per the manufacturer’s guidelines (Miltenyi, Cat:130-109-398). Samples were then incubated with CD11B-microbeads (Miltenyi, Cat: 130-097-142) as per manufacturer’s guidelines, then washed with Running Buffer (Miltenyi, Cat: 130-091-221) and centrifuged at 500×*g* for 5 min. Pellets were resuspended with 2 mL Running Buffer and subjected to the autoMACS Pro Separator (Miltenyi, Cat:130-092-545) “Possel” selection protocol. Positive fractions were collected, centrifuged at 500×*g* for 5 min and then re-suspended in 200 μL Qiazol lysis reagent (Qiagen, Cat: 79306) and flash frozen until RNA was isolated.

### Tissue harvest and RNA isolation

Mice were anesthetized with Ketamine and Xylazine. The spinal column was rapidly removed, and the lumbar spinal cord was retrieved. Lumbar spinal cords were flash frozen and stored at − 80 °C until processed. Frozen tissue was kept on dry ice until homogenized in Qiazol reagent (Qiagen Inc, Cat: 79306). Crude homogenate was mixed with chloroform, at a ratio of 70 μL chloroform per 350 μL homogenate, to facilitate removal of lipids, and allowed to equilibrate at room temperature for 2 min. Samples were centrifuged at 13,000×*g* for 15 min at 4 °C. The aqueous phase containing the RNA was transferred to a new tube. Then the following steps were completed as detailed by the manufacturer’s instructions using an RNeasy Mini Kit (Qiagen, Cat: 74104) with on-column DNAse digestion (Qiagen, Cat:79254). RNA concentration was determined via a NanoDrop spectrophotometer (ThermoFisher, Model: ND-1000). RNA integrity (RIN) for RNA-sequencing samples was measured using RNA 6000 Pico Kit in a 2100 Bioanalyzer (Agilent).

### Real-time quantitative polymerase chain reaction

One microgram of RNA was used with the iScript cDNA synthesis kit (BioRad, Cat:1708890) as per the manufacturer’s instructions to generate cDNA. Taqman gene expression assays were used to evaluate *Ager* (Life Technologies, Cat: Mm01134790_g1), *18s* rRNA (Applied Biosystems, Cat:4310893E), *Hprt* (Life Technologies, Cat:Mm03024075_m1), *Il1a* (Life Technologies, Cat:Mm00439620_m1), and *Malat1* (Life Technologies, Cat: Mm01227912_s1) levels using the Taqman Fast Universal PCR Master Mix (Life Technologies, Cat: 4367846) with the 7500 Fast Real-Time PCR System (Applied Biosystems, Cat: LS4351106). Expression values were calculated using the ΔΔCt method relative to *18s* rRNA or *Hprt*.

### Cytokine array

Five hundred nanograms of RNA per biological replicate (1.5 μg total) for each condition was pooled before proceeding with the mouse cytokine cDNA plate array (Signosis, Cat: AP-1141) as per the manufacturer’s instructions. Resulting luminescence was quantified using a SpectraMax M5 microplate reader (Molecular Devices, San Jose, CA) and normalized to *18s rRNA*.

### Murine RNA sequencing

High-quality lumbar spinal cord RNA samples, as confirmed by Bioanalyzer with RIN values ranging from 7.9 to 8.7, free of DNase and RNase were prepared for sequencing using the TruSeqStranded mRNA library prep kit (Illumina, Cat: 20020594) and the NovaSeq 6000 SP Reagent Kit v1.5 (Illumina, Cat: 20028401). High-throughput RNA sequencing (RNA-seq) was completed using an Illumina NovaSeq 6000 sequencer performed by the NYU Grossman School of Medicine Genome Technology Core.

### Murine RNA-seq data analysis

The resulting 49–61 M read pairs per sample were processed following standard quality control practices to remove low-quality reads and adapter sequence contamination (~ 15% of reads) [47–49]. Remaining high-quality read pairs were aligned to the mouse genome (mm10) using STAR 2.7.3a with a mean of 88% uniquely mapping [50]. Read pair counts per gene were summed with the featureCounts function in subread 1.6.3 using the GENCODE M25 annotation release [50–53]. Read counts were normalized using the trimmed mean of M values (TMM) method in edgeR 3.24.3 within the R environment 3.5.1 [54–56]. Differential expression was analyzed using edgeR via the exactTest function [55, 57]. Significantly differentially expressed genes were determined by a cut-off of adjusted *p* value < 0.05 and subjected to over representation analysis of KEGG pathways using the R package clusterProfiler v3.16.1 [58–61]. Significantly differentially expressed genes with false-discovery rate (FDR) < 0.05 and corresponding log_2_ fold changes were used as input for all ingenuity pathway analysis (IPA) including graphical summary, canonical pathways, upstream regulators, and causal network analyses (QIAGEN Inc.) [62]. Adjustments were made for multiple testing to control the FDR at 0.05 [63].

### Human RNA-seq data analysis

Raw RNA-seq data of cervical spinal cord and de-identified metadata were obtained from:The Target ALS Multicentered Postmortem Tissue Core, the New York Genome Center for Genomics of Neurodegenerative Disease, Amyotrophic Lateral Sclerosis Association, and TOW Foundation (www.targetals.org). Illumina paired-end 100-bp read data were downloaded from TargetALS (mean of 43 M reads per sample) and processed following standard quality control practices to remove low-quality reads and adapter sequence contamination (~ 4% of reads) [47–49]. Remaining high-quality read pairs were aligned to the human genome (hg19) using STAR 2.6.1d with a mean of 95% uniquely mapping. Read pair counts per gene were summed with the featureCounts function in subread 1.6.3 using the GENCODE 30 annotation release [50–53]. Only samples with RIN values of ≥ 6 were used for all analyses. Read counts were normalized using the TMM method on log_2_ counts per million in edgeR 3.24.3 within the R environment 3.5.1 [54–56]. Differential expression was analyzed using generalized linear models via edgeR, with either (1) diagnosis of ALS spectrum motor neuron disease (*n* = 76) or non-neurological control (*n* = 11) as categorical variables; or (2) *AGER* as a continuous variable in ALS patients (*n* = 76) to control and test for differential expression related to diagnosis or increasing *AGER* expression while controlling for sex and testing for any potential interactions within the model [64]. Significant differentially expressed genes with log_2_ fold changes with absolute values of ≥ 0.5 used as the input for evaluation of overrepresentation of KEGG gene sets and for all IPA analyses as per the murine data analysis described above. Competitive gene set testing via CAMERA, and rotational gene set testing via ROAST were both completed using edgeR 3.24.3 to evaluate KEGG and Gene Ontology (GO) gene sets [55, 57–60, 65–68]. Testing for linear correlation was completed by first normalizing *AGER* expression using the TMM method and generating counts per million mapped reads (CPM) with edgeR 3.24.3 as above. A linear model was generated with normalized *AGER* CPM as the predictor of either age at death/tracheostomy or age at onset within the base R environment 3.5.1.

### Statistics

Data are shown as mean ±SEM or as indicated. Normality of the data was assessed using the Shapiro-Wilk’s normality test. If normality assumption was met, data were subsequently evaluated by independent two-sample *t* tests, two-way ANOVA with post-hoc Tukey’s test, one way ANOVA with post-hoc Holm-Šídák multiple comparisons test, or mixed effects analysis with Geisser-Greenhouse correction with post-hoc Holm-Sidak’s multiple comparisons test, as indicated. Non-parametric Mann–Whitney tests were implemented instead to assess differences if normality assumption was violated. Survival data were visualized by Kaplan–Meier curve and the Logrank test (Mantel–Cox) was used to evaluate differences in the survival distributions between groups. All analyses were performed with GraphPad Prism 9 (GraphPad Software, San Diego, CA) and R 3.6.1 using R package “nlme” [56, 69]. *p* values < 0.05 were used to denote statistical significance.

## Results

### Evaluation of *AGER* expression in human amyotrophic lateral sclerosis spinal cord

Previous studies have documented increased RAGE expression in human ALS spectrum motor neuron disease (ALS) spinal cord [25, 26]; however, detailed assessments of roles for RAGE in ALS are lacking [41]. To address this key point, we obtained bulk RNA-seq data from ALS and control patient cervical spinal cord tissue from the Target ALS Multicentered Postmortem Tissue Core, the New York Genome Center for Genomics of Neurodegenerative Disease, Amyotrophic Lateral Sclerosis Association and TOW Foundation. We limited our analysis to RNA-seq samples with RIN ≥ 6. In total, 76 ALS and 11 non-neurological control cervical spinal cord data met these criteria and were eligible for analysis.

**Fig. 1:**
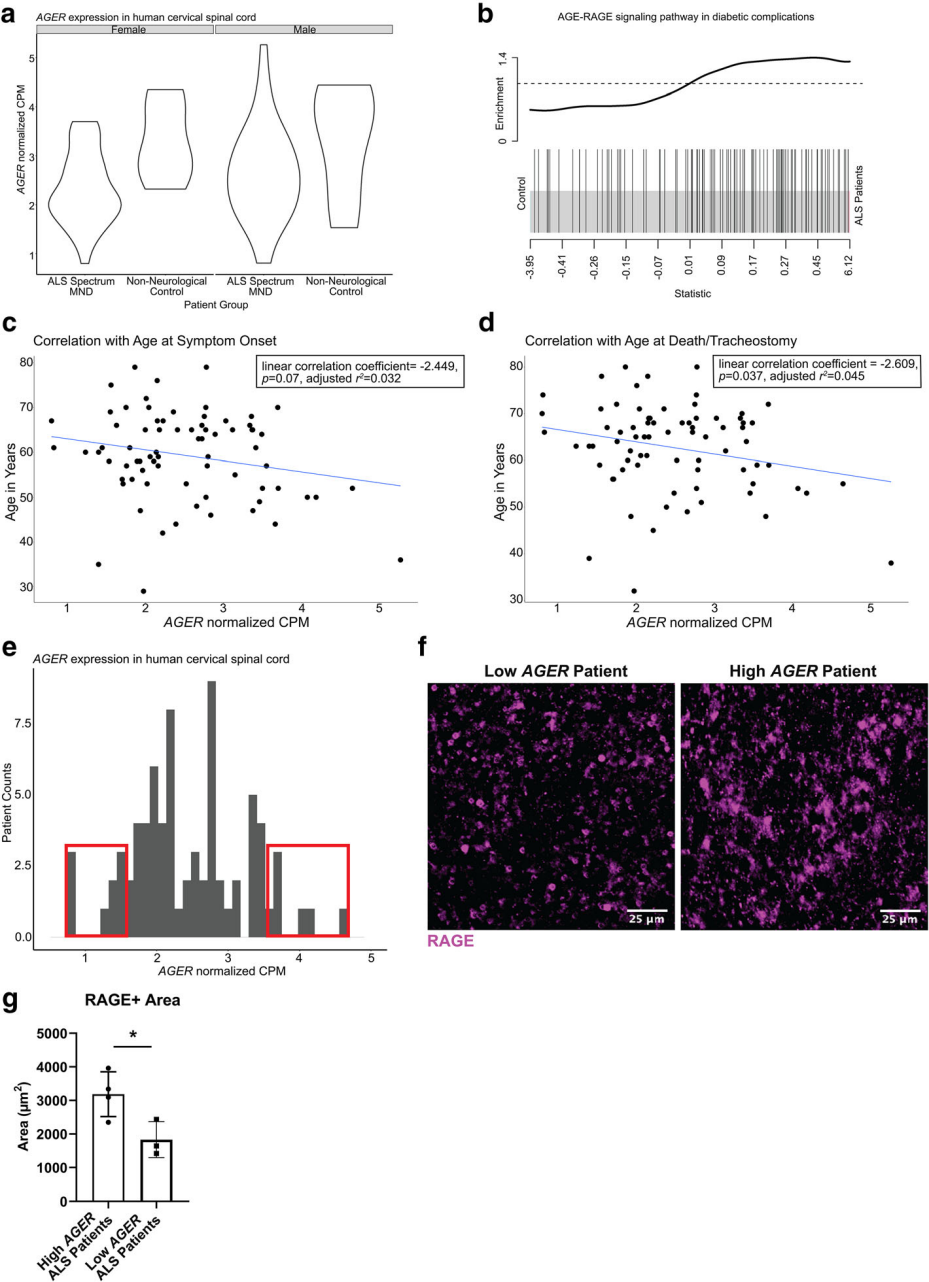
Analysis of human ALS patient RNA-seq data implicates the RAGE pathway in modulating disease*.* **a** Normalized *AGER* counts per million mapped reads across patient groups separated by sex (no significant sex-dependent differences). **b** Barcode plot illustrating gene set enrichment of the “AGE-RAGE signaling pathway in diabetic complications” between ALS patients and non-neurological controls. The snake illustrates the enrichment and upregulation of the gene set in ALS patients. **c** Correlation of *AGER* expression (*x*-axis) and age at onset in years (*y*-axis). Regression line shown. Coefficient = − 2.449, *p* = 0.07, adjusted *r*^2^ = 0.032. **d** Correlation of *AGER* expression (*x*-axis) and age at death/tracheostomy in years (*y*-axis). Regression line shown. Coefficient = − 2.609, *p* = 0.037, adjusted *r*^2^ = 0.045. **e** Histogram of normalized *AGER* expression in ALS patients. Red boxes notate the highest 10% and the lowest 10% of *AGER*-expressing patients. **f** Representative image of regions of interest for RAGE staining in the ventral horn of a high and low *AGER* patient cervical spinal cord. Scale bar: 25 μm. **g** Quantification of RAGE+ area. In **a**: from left to right *N* = 38, 5, 38, 6. In **b**: *N* = 76 ALS patients and *N* = 11 non-neurological control patients. In **c**–**e**: *N* = 76 ALS patients. In **f**, **g**: *N* = 4 high *AGER* and 3 low *AGER* patients. Independent two sample two-sided *t* test. *p = 0.0349

Expression of the gene encoding RAGE, *AGER*, was not significantly different (*FDR* = 0.78) between ALS patients and non-neurological controls (Fig. 1A). However, when we examined gene set differences in expression between the ALS patients and non-neurological controls with rotational gene set testing (ROAST) and competitive gene set testing (CAMERA) analyses, we observed significant enrichment of the “AGE-RAGE signaling pathway in diabetic complications” which was visualized by a barcode enrichment plot for the pathway (Fig. 1B, see Additional Files 1 and 2, Supplemental Table 1.1-1.3). Altogether, these analyses suggest upregulation of the AGE-RAGE signaling pathway gene set in ALS patients relative to control patients.

Further analysis revealed that there was a spectrum of *AGER* expression (normalized counts) in the cervical spinal cord across ALS patients (Fig. 1A). Thus, it was logical to determine if the level of *AGER* correlated with available ALS patient phenotypic metadata. The ALS patients had a median onset of 60.5 years of age (range 32–80), median age at death of 65 years (range 32–80), and a median disease duration (onset to death) of 36mo (range 6–156 months), and the majority had no identifiable family history of ALS (see Additional Files 1 and 2, Supplemental Table1.4). We found normalized *AGER* counts per million (CPM) was negatively correlated with the age at disease onset (coefficient = − 2.449, *p* = 0.07, adjusted *r*^2^ = 0.032) and the age at death or tracheostomy (coefficient = − 2.609, *p* = 0.037, adjusted *r*^2^ = 0.045) in the ALS patients (Fig. 1C, D). We obtained a randomly-selected and available subset of tissue sections from the patients displaying highest vs. lowest 10% of *AGER* RNA values, that is, the extremes (Fig. 1E). We demonstrated that the difference in *AGER* RNA was also present at the protein level (Fig. 1F, G).

We next sought to examine genes and pathways that may be modulated with increasing *AGER* expression in ALS patients. We tested for differential expression using *AGER* as the predictive variable and found that there were many significantly differentially expressed genes associated with *AGER* expression (see Additional Files 1 and 2, Supplemental Table 1.5). Importantly, there were no differential genes related to *AGER* expression that differed significantly by sex. We narrowed subsequent analyses to differential genes with ≥ 0.5 |Log_2_| fold change; it is important to note that Log_2_ fold changes represent the degree to which each gene changes per change in *AGER* expression. KEGG pathway enrichment results indicate upregulation of cell-cell communication and extracellular matrix remodeling pathways with the extent of *AGER* expression (see Additional Files 1 and 2, Supplemental Table 1.6). Ingenuity pathway analysis (IPA) indicated several enriched canonical pathways of genes including hepatic fibrosis/hepatic stellate cell activation (Table 1). Altogether, these data support roles for RAGE in human ALS.

**Table 1:** Overrepresentation analyses dependent on *AGER* expression within ALS patient cervical spinal cord RNA-seq

Ingenuity canonical pathways	FDR	KEGG pathways	FDR
Hepatic fibrosis/hepatic stellate cell activation	6.46E-08	Protein digestion and absorption	3.71E-07
GP6 signaling pathway	2.69E-07	ECM-receptor interaction	2.79E-05
Agranulocyte adhesion and diapedesis	7.08E-03	Cell adhesion molecules	2.27E-02
Apelin liver signaling pathway	7.08E-03	Calcium signaling pathway	4.21E-02
Sperm motility	9.55E-03	PI3K-Akt signaling pathway	4.21E-02
Atherosclerosis Signaling	1.58E-02		

Genes that were input into these analyses were significantly differentially-expressed genes with FDR < 0.05 with absolute log_2_ fold change values of ≥ 0.5. Enriched pathways, FDR < 0.05

**Fig. 2:**
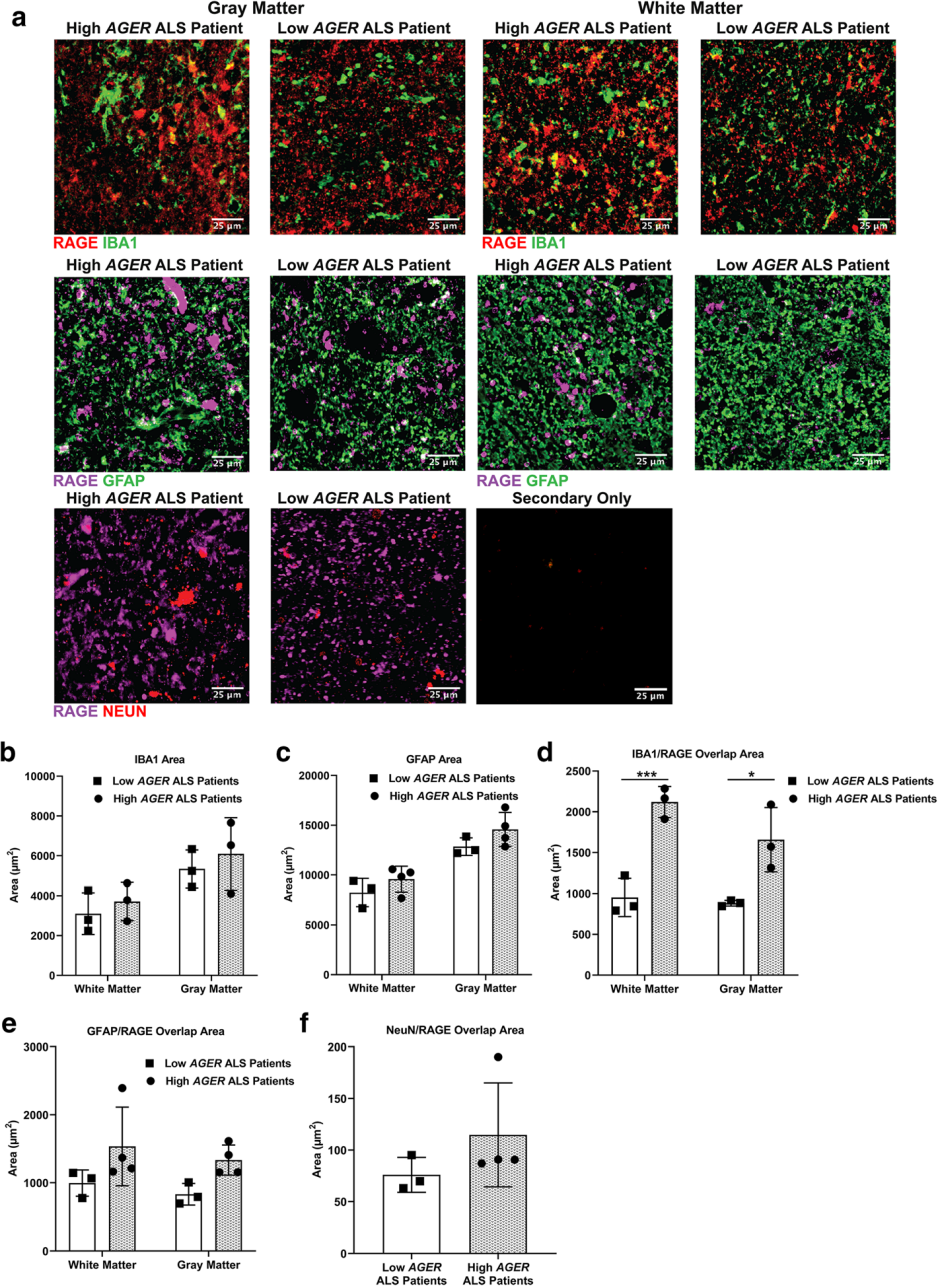
*AGER* expression changes coincide with alterations in overlap of microglia and RAGE protein. **a** Representative images of regions of interest for RAGE, IBA1, GFAP, and NeuN staining in the ventral horn of high and low *AGER* patient cervical spinal cord and anterior white matter. Scale bar: 25 μm. **b** Quantification of IBA1^+^ area. **c** Quantification of GFAP^+^ area. **d** Quantification of overlap of IBA1^+^ RAGE^+^ area. **e** Quantification of overlap of GFAP^+^ RAGE^+^ area. **f** Quantification of overlap of NeuN^+^ RAGE^+^ area. *N* = 4 high *AGER* patients and *N* = 3 low *AGER* patients. Mean *±* SD. In **b**–**e**: two-way ANOVA with post-hoc Tukey’s test. In **f**: Mann-Whitney *U* test. *p = 0.0102, ***p = 0.0009

However, as these analyses are from bulk RNA-seq data of cervical spinal cord tissue, it was not possible to discern which cell type(s) may be expressing RAGE and if this too differs across the spectrum of *AGER*-expressing ALS patients. To address this point, we sought to co-localize RAGE expression with several cell type markers within the subset of patients with the extremes of RAGE expression. We performed a series of immunohistochemistry (IHC) experiments to determine which cell types in the ALS cervical spinal cord were expressing RAGE and if that differed between these ALS patients. We found that there were no differences in microglia (ionized calcium-binding adapter molecule 1, IBA1) or astrocyte (glial fibrillary acidic protein, GFAP) staining area in either the ventral horn gray matter or associated white matter between high and low *AGER* patients (Fig. 2A–C). However, there were significantly higher levels of RAGE overlap with IBA1 in both areas, but not with GFAP in either area, in high vs. low *AGER* patients (Fig. 2A, D, E). Furthermore, there was no difference in the overlap of RAGE with a neuronal marker, neuronal nuclei antigen (NeuN), within the ventral horn between high and low *AGER* patients (Fig. 2A, F). However, additional RAGE signal was detected, which was not localized to any of these cell-type markers and the cellular or extracellular nature remains unclear. These data support the recent spatial transcriptomic analyses that indicated enrichment of the AGE-RAGE pathway in a glia module associated with disease progression in the *SOD1*^*G93A*^ murine ALS model and in a corresponding module in human ALS patient tissue [12]. Altogether, these data suggest that *AGER* expression may be associated with (1) ALS pathology and (2) glial perturbation.

### *SOD1*^*G93A*^ mice exhibit increased RAGE-expressing microglia during pathology progression

**Fig. 3:**
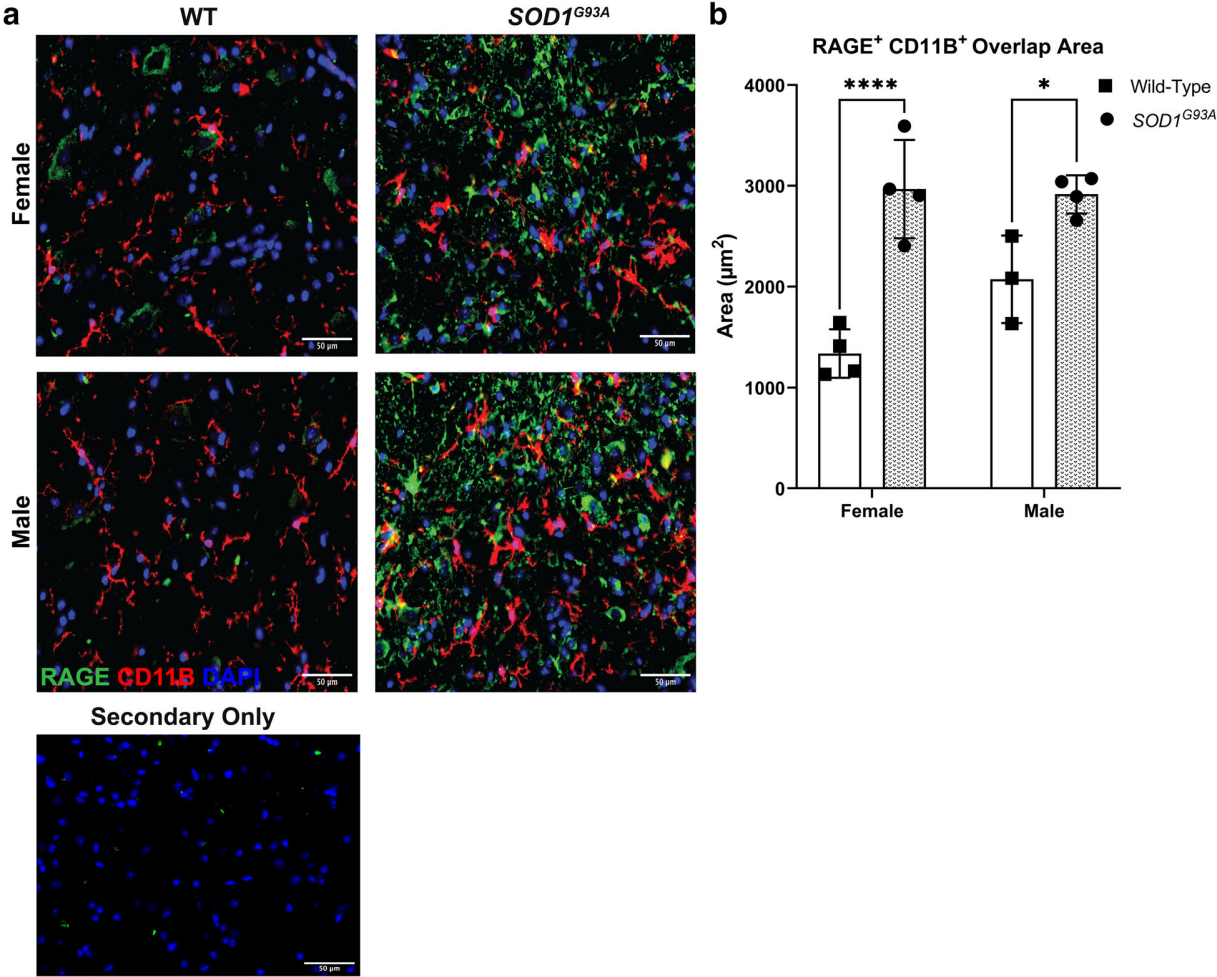
*SOD1*^*G93A*^ mice exhibit increased RAGE^+^ microglia at the age of 120 days. **a** Representative images of CD11B and RAGE staining in the ventral horn of *SOD1*^*G93A*^ mouse lumbar spinal cord. Scale bar: 50 μm. **b** Quantification of overlap of CD11B^+^ RAGE^+^ area. *N* = 3 male WT mice, *N* = 4 male *SOD1*^*G93A*^ mice, *N* = 4 female WT mice, and *N* = 4 female *SOD1*^*G93A*^ mice. Mean *±* SD. Two-way ANOVA with post-hoc Tukey’s test. *p = 0.0192, ****p < 0.0001

Based on these analyses in human ALS and the accumulating evidence that microglia are dysfunctional early in the *SOD1*^*G93A*^ murine model, which may promote/propagate astrocytic and neuronal dysfunction and that RAGE is known to regulate in vitro microglia-like cell responses to several ligands increased in *SOD1*^*G93A*^ mice [25, 33, 34, 70–73], we sought to examine if microglia expressed RAGE in a prototypic murine model of ALS, the *SOD1*^*G93A*^ model. There are currently no mouse models of “sporadic” ALS; however, murine models of familial ALS provide a means to model and test fundamental mechanisms of motor degeneration and reduced survival in these animals vs. their unaffected littermate controls. Hence, we began by performing IHC in *SOD1*^*G93A*^ and WT mice (all in the C57BL/6J background) lumbar spinal cord tissue at age 120 days. The overlap area of RAGE with the myeloid marker, integrin subunit alpha m (CD11B), was higher in *SOD1*^*G93A*^ mice vs. littermate WT control mice (Fig. 3A, B). While RAGE is expressed in multiple cell-types in spinal cord; the findings that RAGE overlap with microglia is increased in *SOD1*^*G93A*^ mice and in a subset of human patients with higher *AGER* expression, alongside the spatial transcriptomic analyses, implicating the AGE-RAGE pathway alteration in glia, suggested it was logical to probe potential roles for microglia RAGE in ALS-like pathology in the *SOD1*^*G93A*^ mouse model.

### Microglia *Ager* deletion extends survival in male *SOD1*^*G93A*^ mice

**Fig. 4:**
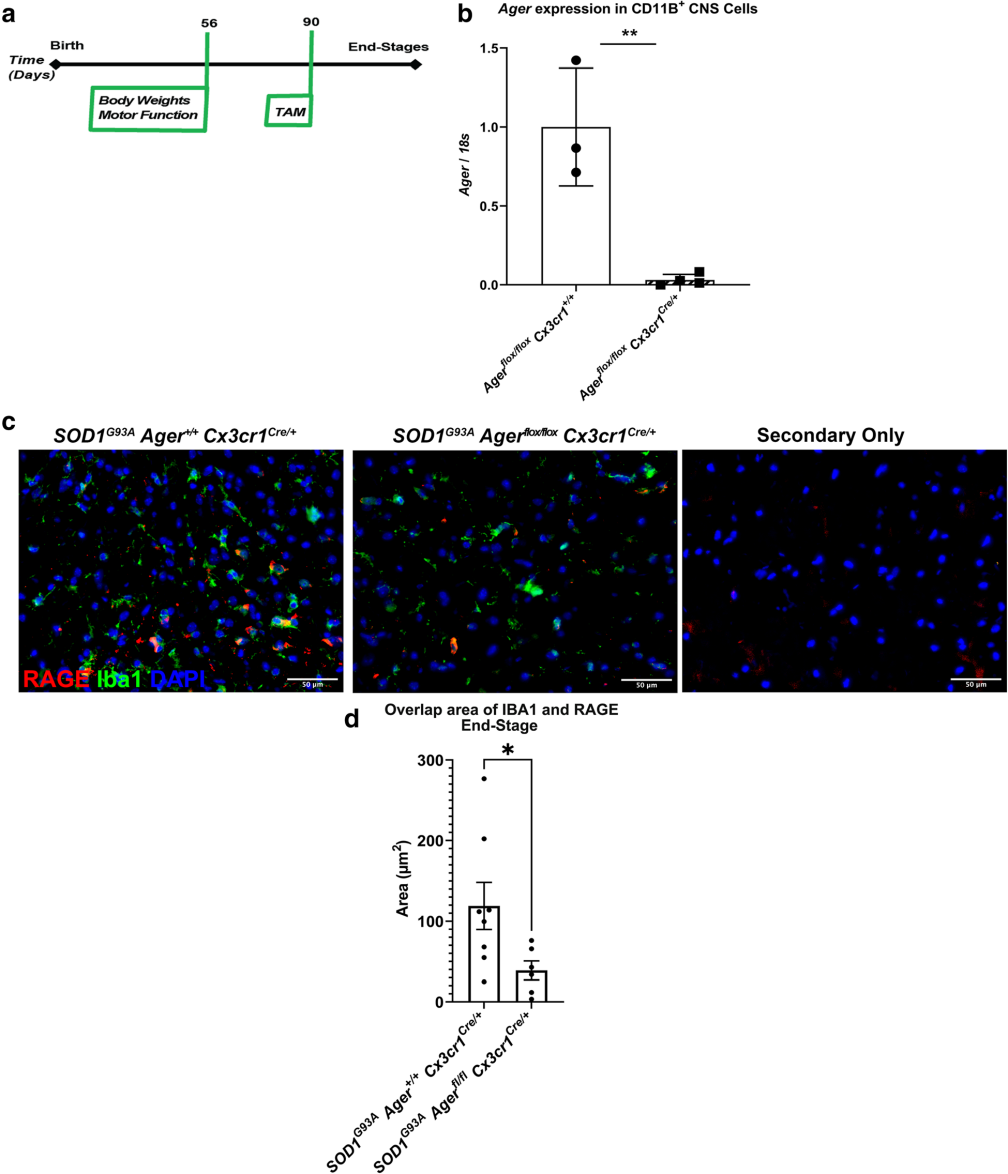
Study design and validation of microglia *Ager* deletion. **a** Timing of Tamoxifen (TAM) administration and behavioral assessments of animals. **b** Quantification of *Ager* transcripts in primary CD11B^+^ isolates by RT-qPCR thirty days after TAM administration. **c** Representative images of IBA1 and RAGE staining in the ventral horn of *SOD1*^*G93A*^ mouse lumbar spinal cord at end-stage. Scale bar: 50 μm. **d** Quantification of IBA1 and RAGE overlap area. Mean *±* SD. In **b**, *N* = 3 *Ager*^fl/fl^ *Cx3cr1*^+/+^ mice and *N* = 4 *Ager*^fl/fl^ *Cx3cr1*^Cre/+^ mice. In **c**, **d**, *N* = 6 *SOD1*^*G93A*^ *Ager*^fl/fl^ *Cx3cr1*^Cre/+^ mice, *N* = 8 *SOD1*^*G93A*^ *Ager*^+/+^ *Cx3cr1*^Cre/+^ mice. Independent two sample two-sided *t* test. **p = 0.0031, *p = 0.0437

As accruing evidence has suggested stage-specific roles for microglia in the context of ALS and other neurodegenerative diseases, we sought to assess whether microglia RAGE affects the pathological progression of *SOD1*^*G93A*^ mice [14]. We employed a tamoxifen (TAM)-inducible model in which microglia expressed *Ager* during development and early life and administered TAM to all mice at the age of 90 days to induce *Ager* deletion in *SOD1*^*G93A*^ *Ager*^fl/fl^ *Cx3cr1*^Cre/+^, and *SOD1*^*G93A*^ *Ager*^+/+^ *Cx3cr1*^Cre/+^ mice to evaluate potential roles for RAGE in the progression of pathology (Fig. 4A). We confirmed knock-down of *Ager* expression in primary CD11B cell isolates from central nervous system (CNS) tissues (Fig. 4B). The area of RAGE overlap with IBA1, but not GFAP, was significantly lower in the *SOD1*^*G93A*^ *Ager*^fl/fl^ *Cx3cr1*^Cre/+^ lumbar spinal cord tissue relative to Cre-expressing controls at the end of the study, suggesting *Ager* knock-down in microglia was maintained (Fig. 4C, D, Supplemental Figure 1A-B). The area of RAGE overlap with the pan neuronal marker microtubule-associated protein 2 (MAP2) at the age of 120 days did not differ between the two genotypes, suggesting that neuronal *Ager* was not modulated by this approach in this study (Supplemental Figure 1C-D).

**Fig. 5:**
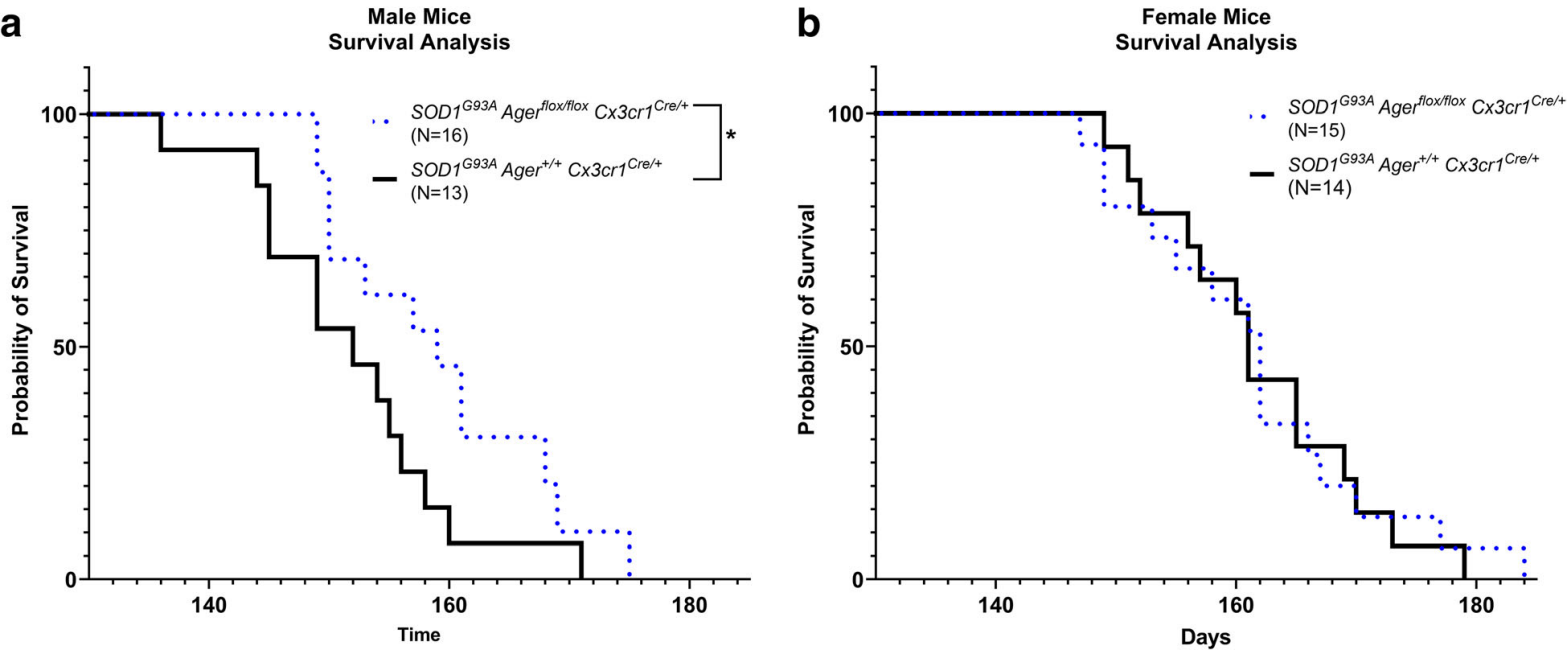
Microglia *Ager* deletion exerts sex-dependent effects on survival in *SOD1*^*G93A*^ mice. **a** Kaplan–Meier estimates of percent of male mice surviving plotted vs. age (days). *N* = 16 *SOD1*^*G93A*^ *Ager*^fl/fl^ *Cx3cr1*^Cre/+^ mice, and *N* = 13 *SOD1*^*G93A*^ *Ager*^+/+^ *Cx3cr1*^Cre/+^ mice. **b** Kaplan–Meier estimates of percent of female mice surviving plotted vs. age (days). *N* = 15 *SOD1*^*G93A*^ *Ager*^fl/fl^ *Cx3cr1*^Cre/+^ mice, and *N* = 14 *SOD1*^*G93A*^ *Ager*^+/+^ *Cx3cr1*^Cre/+^ mice. The Logrank test was performed to compare the survival distributions between groups. *p = 0.0496

As *Cx3cr1* Cre-expressing mice, regardless of the administration of TAM, or not, are heterozygous for the *Cx3cr1* locus, we evaluated *SOD1*^*G93A*^ *Ager*^+/+^ *Cx3cr1*^Cre/+^ (microglia *Ager*-expressing, *Cx3cr1* Cre-expressing) and *SOD1*^*G93A*^ *Ager*^fl/fl^ *Cx3cr1*^Cre/+^ (microglia *Ager* deficient, *Cx3cr1*-Cre expressing) mice [37]. As expected, there were no differences in the age of disease onset between any groups, as defined as the age at which the mouse reached maximum weight (Supplemental Figure 2A-B, see Additional file 1; *p* > 0.05). We found that male *SOD1*^*G93A*^ *Ager*^fl/fl^ *Cx3cr1*^Cre/+^ mice displayed significantly longer survival relative to *SOD1*^*G93A*^ *Ager*^+/+^ *Cx3cr1*^Cre/+^ mice, median lifespan of 159 (range 149–175 days) days vs. 152 days (range 136–171 days), respectively (Fig. 5A; *p* < 0.05). We did not observe differences in lifespan between the female mice groups (Fig. 5B; *p* > 0.05). Altogether, these data suggested that microglia RAGE may contribute to pathology progression in male *SOD1*^*G93A*^ mice.

### Male *SOD1*^*G93A*^*Ager*^fl/fl^*Cx3cr1*^*Cre*/+^ mice displayed reduced pathology during disease progression

**Fig. 6:**
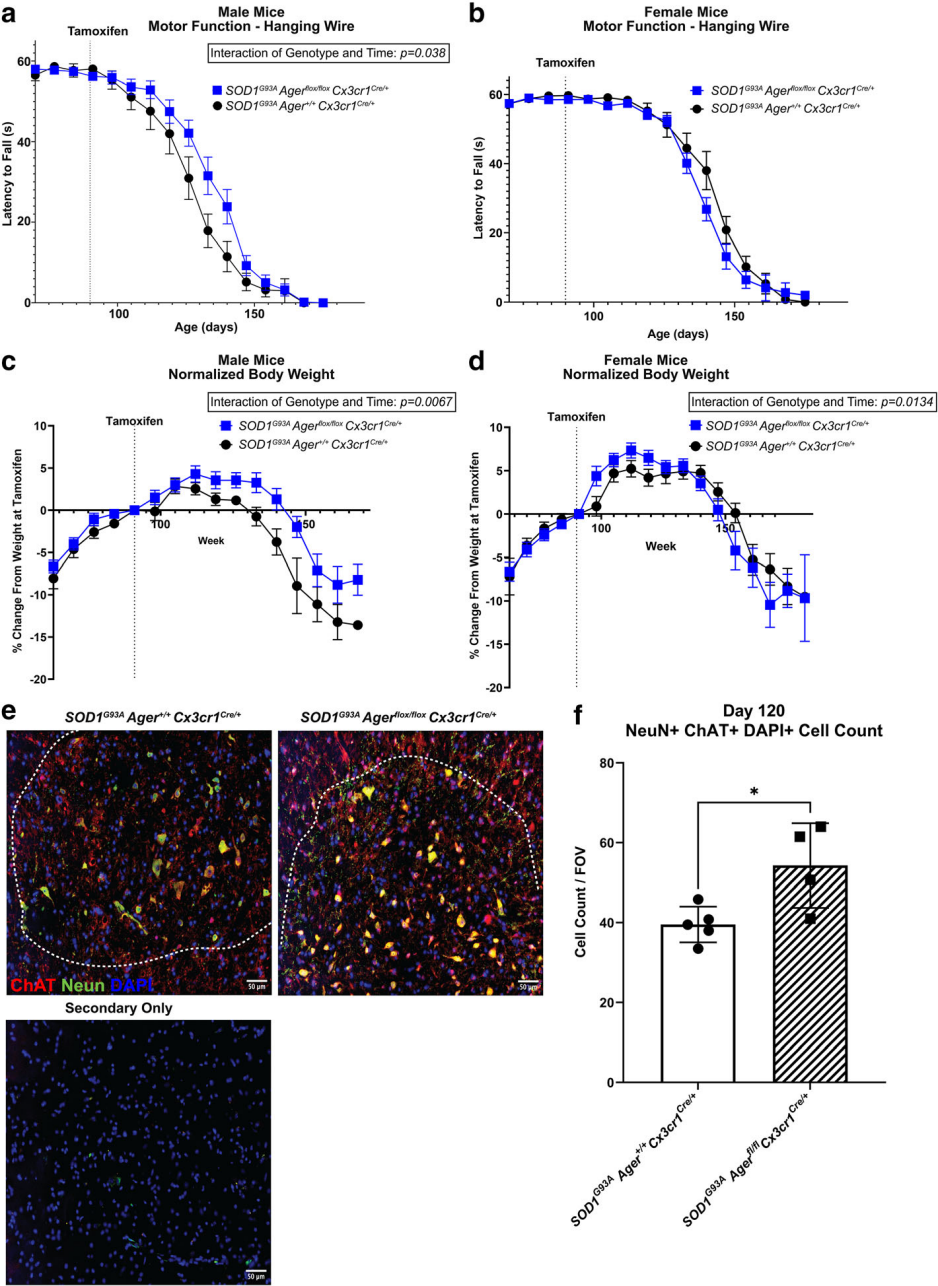
*SOD1*^*G93A *^mice with microglia *Ager* deletion exhibit sex-dependent effects on pathology. **a** Latency to fall as measured by hanging wire test in male mice. **b** Latency to fall as measured by hanging wire test in female mice. **c** Normalized body weight to weight at tamoxifen administration of male mice. **d** Normalized body weight to weight at tamoxifen administration of female mice. Dashed lines indicate age at tamoxifen administration. Mean *±* SEM. **e** Representative images of NeuN and DAPI staining in the lumbar spinal cord ventral horn. Scale bar: 50 μm. **f** Quantification of NeuN^+^ DAPI^+^ cell number. Mean *±* SD. In **a**, **c** (male mice): *N* = 16 *SOD1*^*G93A*^ *Ager*^fl/fl^ *Cx3cr1*^Cre/+^ mice, and *N* = 13 *SOD1*^*G93A*^ *Ager*^+/+^ *Cx3cr1*^Cre/+^ mice. In **b**, **d** (female mice): *N* = 15 *SOD1*^*G93A*^ *Ager*^fl/fl^ *Cx3cr1*^Cre/+^ mice, and *N* = 14 *SOD1*^*G93A*^ *Ager*^+/+^ *Cx3cr1*^Cre/+^ mice. In **e**, **f** (male mice): *N* = 4 *SOD1*^*G93A*^ *Ager*^fl/fl^ *Cx3cr1*^Cre/+^ mice, and *N* = 5 *SOD1*^*G93A*^ *Ager*^+/+^ *Cx3cr1*^Cre/+^ mice. In **a**–**d**: independent two-sample two-sided *t* test was used to assess the group difference at each time point. Mixed effects analysis was utilized to evaluate effects of genotype, time, and the interaction between genotype and time. In **a**: genotype*time interaction effect: *p* = 0.0378. In **c**: genotype effect: *p* = 0.0157, genotype*time interaction effect: *p* = 0.0067. In **d**: genotype*time interaction effect *p* = 0.0134. In **f**: independent two sample two-sided *t* test. In **f**: *p = 0.0224

We next investigated if microglia *Ager* contributed to the progressive motor function decline experienced by *SOD1*^*G93A*^ mice [4, 5, 38]. Mixed effects analysis of motor function data indicated a significant interaction between time and genotype in male but not female mice (*p* = 0.038) suggesting that male but not female *SOD1*^*G93A*^ *Ager*^fl/fl^ *Cx3cr1*^Cre/+^ mice had time-dependent protection in motor function (Fig. 6A, B). However, there were no significant differences in motor function at any one time point after multiple corrections in either male or female mice (Fig. 6A, B).

As *SOD1*^*G93A*^ mice experience pronounced weight loss with disease progression, we next examined if microglia *Ager* expression modulated this weight loss [4, 5, 38]. Mixed effects analysis of body weight data normalized to weight at tamoxifen administration indicated significant interactions between time and genotype in male and female mice (*p* = 0.0067, *p* = 0.0134 respectively*)* suggesting that in *SOD1*^*G93A*^ *Ager*^fl/fl^ *Cx3cr1*^Cre/+^ mice there are time dependent alterations in body weight (Fig. 6C, D). However, there were no significant differences in normalized body-weight measures at any single time point after multiple corrections in male or female mice (Fig. 6C, D). As female mice did not display any significant differences in survival, or motor function, these mice were not investigated further (Figs. 5B and 6B, D).

While no significant differences in motor function or body weight were noted at any single time point, there were time-dependent reductions in the rate of motor function decline and weight in male *SOD1*^*G93A*^ *Ager*^fl/fl^ *Cx3cr1*^Cre/+^ mice suggesting a slower rate of disease progression. This led us to consider the possibility that microglia *Ager* deletion may have affected the numbers of surviving motor neurons within the lumbar ventral horn. We examined the number of surviving motor neurons by IHC at the age of 120 days in male mice and found significantly higher numbers of motor neurons, as labeled by NeuN, DAPI, and choline acetyltransferase (ChAT), in the lumbar ventral horn in the *SOD1*^*G93A*^ *Ager*^fl/fl^ *Cx3cr1*^Cre/+^ mice relative to the Cre-recombinase expressing controls (Fig. 6E, F)*.* Altogether, these data suggest that deletion of microglia *Ager* may reduce/delay neuron death in male *SOD1*^*G93A*^ mice. These findings led us to perform additional experiments to provide insight into the mechanisms by which this protection may occur.

### Microglia *Ager* deletion does not impact skeletal muscle macrophages

Next, we considered that deletion of *Ager* from *Cx3cr1* expressing cells at 90 days of age may have modulated skeletal muscle pathology either by direct or indirect effects on skeletal muscle macrophages and/or peripheral monocyte-derived macrophages. We evaluated end-stage and day 120 gastrocnemius muscle for macrophage content, as labeled by F4/80 and CD68, which revealed no differences between groups at either time point (Supplemental Figure 3A-D, 4A-D). Altogether, these data suggest the beneficial effects of microglia *Ager* reduction were likely restricted to the nervous system.

### Male *SOD1*^*G93A*^*Ager*^*fl*/fl^*Cx3cr1*^*Cre*/+^ mice lumbar spinal cord exhibits transcriptomic alterations suggestive of improved homeostatic function

**Fig. 7:**
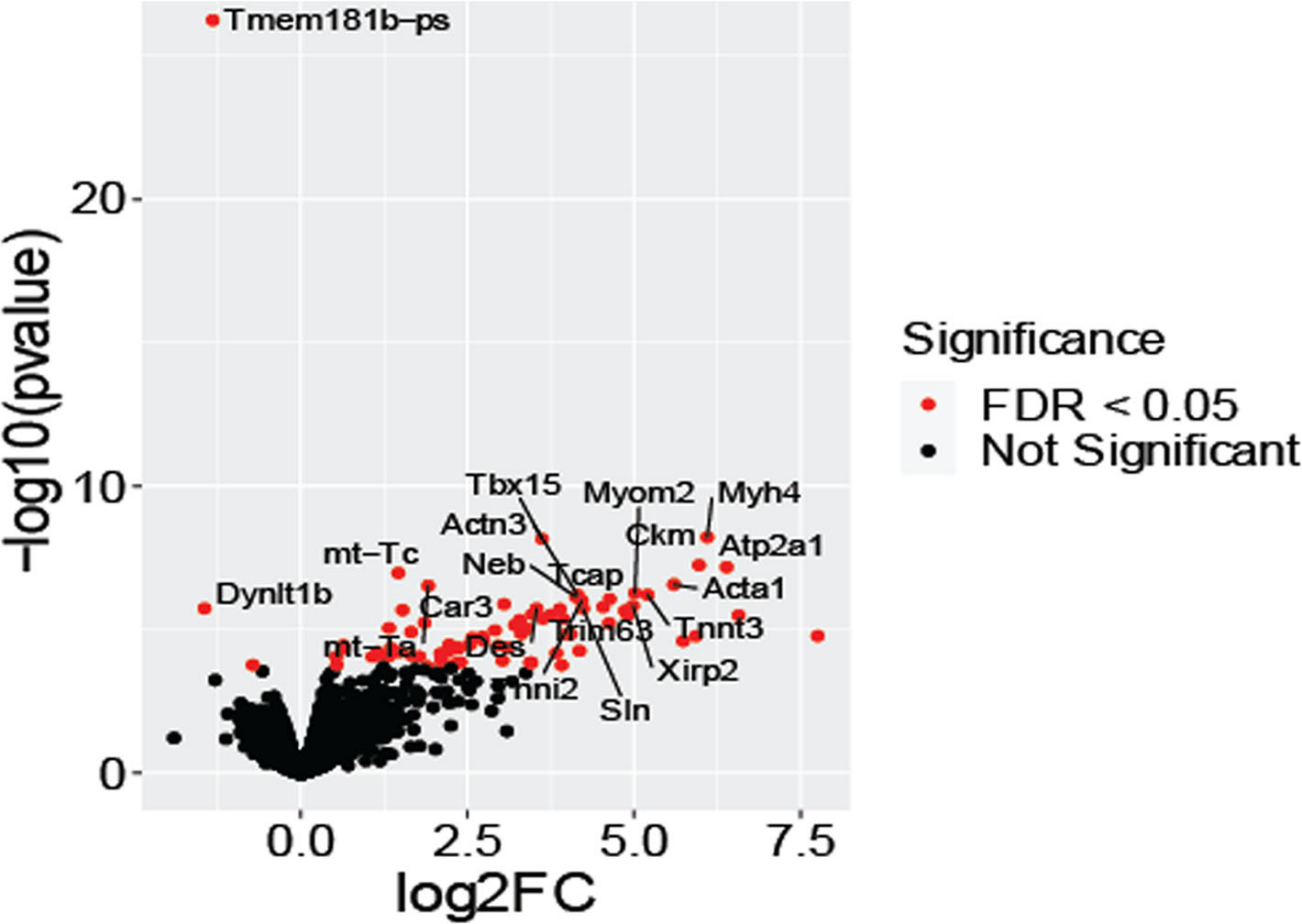
Differential expression analysis between male *SOD1*^*G93A*^ mice with microglia devoid of *Ager *and Cre-expressing controls. Volcano plot displaying differentially expressed genes between *SOD1*^*G93A*^ *Ager*^fl/fl^ *Cx3cr1*^Cre/+^ and *SOD1*^*G93A*^ *Ager*^+/+^ *Cx3cr1*^Cre/+^ mice. The *y*-axis is –log_10_(*p* value) and *x*-axis is the Log_2_ fold change of each gene analyzed. Red colored genes are significantly differentially expressed with an FDR < 0.05. The top twenty significant genes are labeled. *N* = 4 independent mice/group

Hence, to uncover cell intrinsic and cell-cell communication pathway mechanisms underlying the benefits of microglia *Ager* deletion in male *SOD1*^*G93A*^ mice, we performed RNA-seq on isolated lumbar spinal cord tissue from male *SOD1*^*G93A*^ *Ager*^fl/fl^ *Cx3cr1*^Cre/+^ mice and *SOD1*^*G93A*^ *Ager*^+/+^ *Cx3cr1*^Cre/+^ mice at humane endpoint (end-stage). We identified 78 differentially expressed genes between the two genotypes (Fig. 7, Supplemental Table 1.7, see Additional Files 1 and 2). Overrepresentation analysis of the differential gene list indicated enrichment for several KEGG Pathways (Table 2, Supplemental Table 1.8, see Additional Files 1 and 2). Enriched KEGG pathway gene sets included “Cardiac muscle contraction,” “Calcium signaling pathway,” “PPAR signaling pathway,” “Regulation of actin cytoskeleton,” and “Cholesterol metabolism”. Furthermore, canonical pathway analysis using IPA indicated enrichment of several pathways within the differential gene list including “Calcium signaling,” “Actin cytoskeleton signaling,” and “Integrin signaling” among others (Table 2, Supplemental Table 1.9, see Additional Files 1 and 2) [62]. Altogether, these data suggest genotype-dependent effects on cell-cell crosstalk mechanisms, as exemplified by alterations of lipid, metabolic, and integrin signaling gene sets.

**Table 2:** Overrepresentation analyses of differentially expressed genes between *SOD1*^G93A^ *Ager*^fl/fl^ *Cx3cr1*^Cre/+^ and *SOD1*^G93A^ *Ager*^+/+^ *Cx3cr1*^Cre/+^ mice

Ingenuity canonical pathways	FDR	KEGG pathway	FDR
Calcium signaling	1.82E-05	Cardiac muscle contraction	2.02E-05
Actin cytoskeleton signaling	8.91E-05	Hypertrophic cardiomyopathy	2.02E-05
Cellular effects of sildenafil (Viagra)	1.07E-04	Dilated cardiomyopathy	2.02E-05
Epithelial adherens junction signaling	7.24E-04	Calcium signaling pathway	1.2E-3
ILK signaling	7.24E-04	Apelin signaling pathway	1.6E-3
RhoA signaling	1.41E-03	Adrenergic signaling in cardiomyocytes	2.39E-2
Protein kinase A signaling	1.41E-03	Arrhythmogenic right ventricular cardiomyopathy	2.61E-2
Tight junction signaling	2.82E-03	PPAR signaling pathway	3.44E-2
Hepatic fibrosis/hepatic stellate cell activation	2.95E-03	Focal adhesion	4.35E-2
Agranulocyte adhesion and diapedesis	3.47E-03		

Significantly differentially expressed genes, FDR < 0.05 were used as input into each analysis. Top ten enriched pathways, FDR < 0.05

To further address this concept, we analyzed potential causal networks that may be modulated in our dataset, which could explain the observed transcriptomic changes [62]. As *Ager* was deleted solely in *Cx3cr1*-expressing cells, we limited our analysis to potential cytokines that could originate from microglia and cause transcriptomic alterations across multiple cell types which would be present in the bulk RNA-sequencing data. We identified several significant putative causal networks belonging to numerous cytokine families, including interleukin-1 (IL1), interleukin-3, interferon-α (IFN-α), and interferon-β (IFN-β) (Table 3, Supplemental Table 1.10, see Additional Files 1 and 2).

**Table 3:** Causal network analysis utilizing *SOD1*^G93A^ *Ager*^fl/fl^ *Cx3cr1*^Cre/+^ and *SOD1*^G93A^ *Ager*^+/+^ *Cx3cr1*^Cre/+^ differentially expressed genes

Cytokine	Z-Score (activation score)	FDR
CXCL1	3.464	5.48E-05
CXCL9	3.317	1.24E-04
IL3	3.286	1.98E-08
TSLP	3.266	5.78E-12
IL5	2.985	4.36E-07
IFNA1/IFNA13	2.837	4.26E-08
IL22	2.828	4.09E-06
CD70	2.828	8.82E-05
IL1A	2.4	9.75E-06
IL15	1.569	9.70E-08
WNT1	1.414	6.43E-12
IFNB1	1.183	1.29E-11
TNFSF10	0.365	8.95E-09
IL6	0.333	2.89E-04
TNFSF12	-1	9.70E-04

Results of IPA predicted causal network regulators, which are labeled as “cytokine”. Positive values indicate predicted activation in *SOD1*^G93A^ *Ager*^+/+^ *Cx3cr1*^Cre/+^ mice

To evaluate potential roles of microglia RAGE in mediating cytokine expression changes, we turned to an in vitro system. We first conducted a commercially available cytokine screen to evaluate RAGE-dependent effects of RAGE ligand carboxymethyllysine (CML)-AGE, a ligand increased in *SOD1*^*G93A*^ spinal cords and in human patient tissues, on BV2 microglia-like cells (Supplemental 5A) [25, 28, 29, 46]. An interesting candidate discovered by this approach was *Il1a*, as this was also predicted by the RNA-seq causal network analysis (Supplemental Figure 5A, Table 3, Supplemental Table 1.10). In fact, in validation experiments, CML-AGE significantly induced *Il1a* expression, which was significantly reduced by pre-treatment with a RAGE inhibitor in BV2 cells (Supplemental Figure 5B) [32]. Furthermore, lentiviral transduction of BV2 cells with short hairpin RNA to significantly reduce *Ager* expression in BV2 cells prevented the CML-AGE associated increase in *Il1a* expression (Supplemental Figure 5C-D). Interestingly, expression of *Malat1*, a differentially expressed inflammation-associated lncRNA in the microglia *Ager*-deficient spinal cord, exhibited a RAGE-dependent increase in BV2 cells in response to CML-AGE (Supplemental Figure 5E, Supplemental Table 1.7) [74].

Collectively, these results point to significant modulation of intrinsic microglia inflammation. In one or more cell types, prompted by microglia *Ager* deletion, fundamental changes in general cellular health are observed, as exemplified by alterations in actin cytoskeleton and calcium related pathways, both of which would be predicted to fundamentally alter cell intrinsic and intercellular communication properties in the spinal cord. To verify the implications of these transcriptomic alterations, we examined these points specifically in end-stage tissues.

### Microglia *Ager* deletion reduces the accumulation of damage-associated microglia in male *SOD1*^*G93A*^ mice

**Fig. 8:**
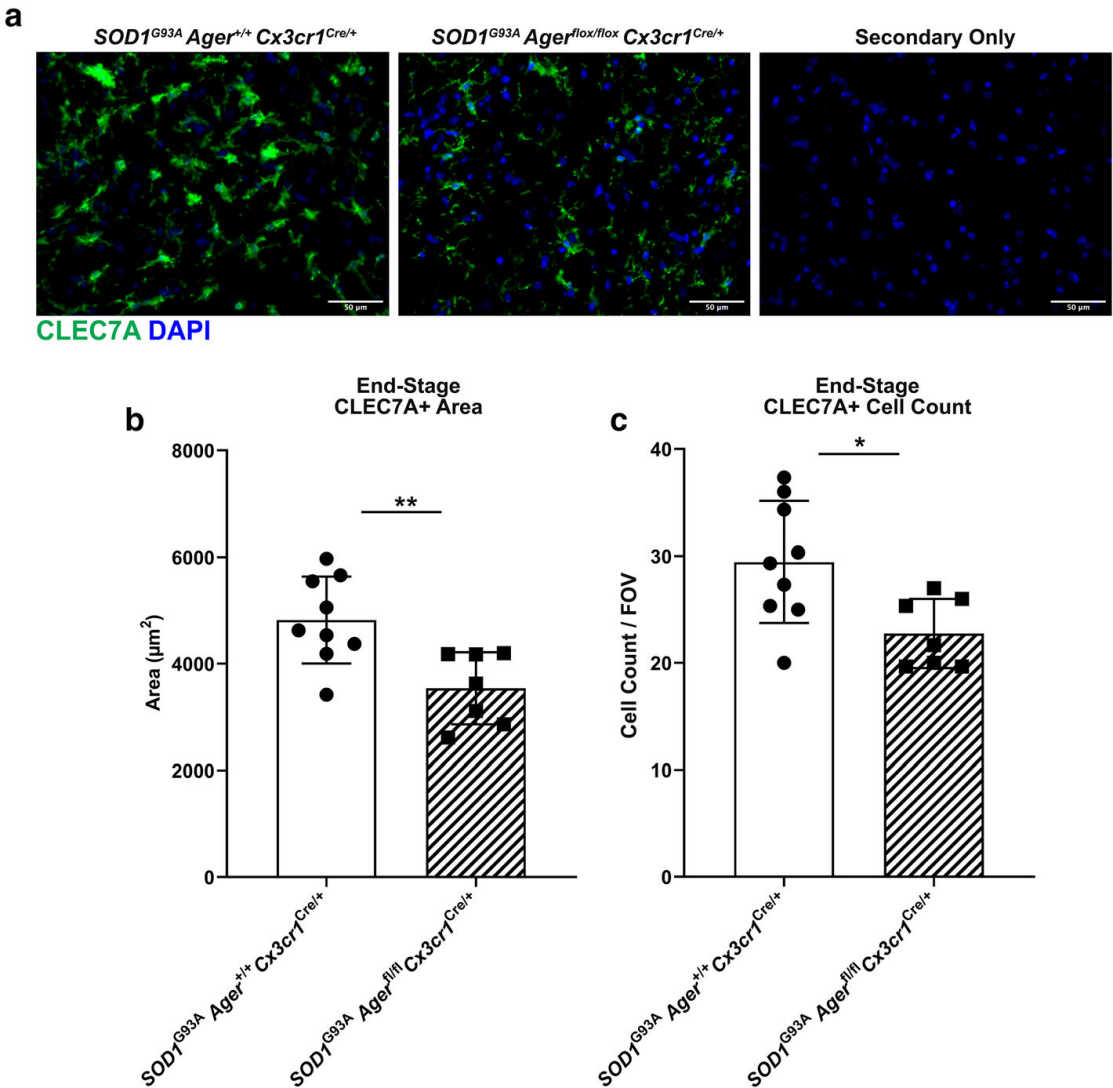
Microglia *Ager* deletion in male *SOD1*^*G93A*^ mice reduces the accumulation of disease-associated microglia at end-stage*.* **a** Representative images of CLEC7A staining in the lumbar spinal cord ventral horn of the indicated mouse groups. Scale bar: 50 μm. **b** Quantification of CLEC7A^+^ area. **c** Quantification of CLEC7A^+^ DAPI^+^ cell number. Mean *±* SD. *N* = 7 *SOD1*^*G93A*^ *Ager*^fl/fl^ *Cx3cr1*^Cre/+^ mice and *N* = 9 *SOD1*^*G93A*^ *Ager*^+/+^ *Cx3cr1*^Cre/+^ mice. Independent two sample two-sided *t* test. In **b**, **p < 0.0048. In **c**, *p = 0.0154

The RNA-seq data analysis suggested that microglia *Ager* deletion resulted in reduction in IL1 and IFN signaling (Table 3), both of which were previously suggested to be dysfunctional in ALS models and in patients [75–79]. In fact, in vitro experiments supported microglia RAGE affecting *Il1a* and *Malat1* expression (Supplemental Figure 3). Beyond the number and density of microglia, their specific gene expression patterns aid in designation of these cells as pro-damage vs. homeostatic phenotype. Specifically, the c-type lectin domain containing 7a protein (CLEC7A) is an established marker of pro-damage/disease-associated microglia, which has been shown to be highly upregulated in *SOD1*^*G93A*^ microglia [23]*.* By IHC, at end-stage, we found that there was a significant reduction in CLEC7A^+^ area, and CLEC7A^+^ cell number in *SOD1*^*G93A*^ *Ager*^fl/fl^ *Cx3cr1*^Cre/+^ vs. *SOD1*^*G93A*^ *Ager*^+/+^ *Cx3cr1*^Cre/+^ mice (Fig. 8A–C), suggesting that an attenuated or altered DAM phenotype was induced by deletion of microglia *Ager*. These key findings raise the possibility that RAGE contributes to a cell-intrinsic transition of microglia from a homeostatic to a dysfunctional phenotype.

### Microglia *Ager* deletion reduces accumulation of reactive astrocytes at the end-stage of disease in *SOD1*^*G93A *^mice

**Fig. 9:**
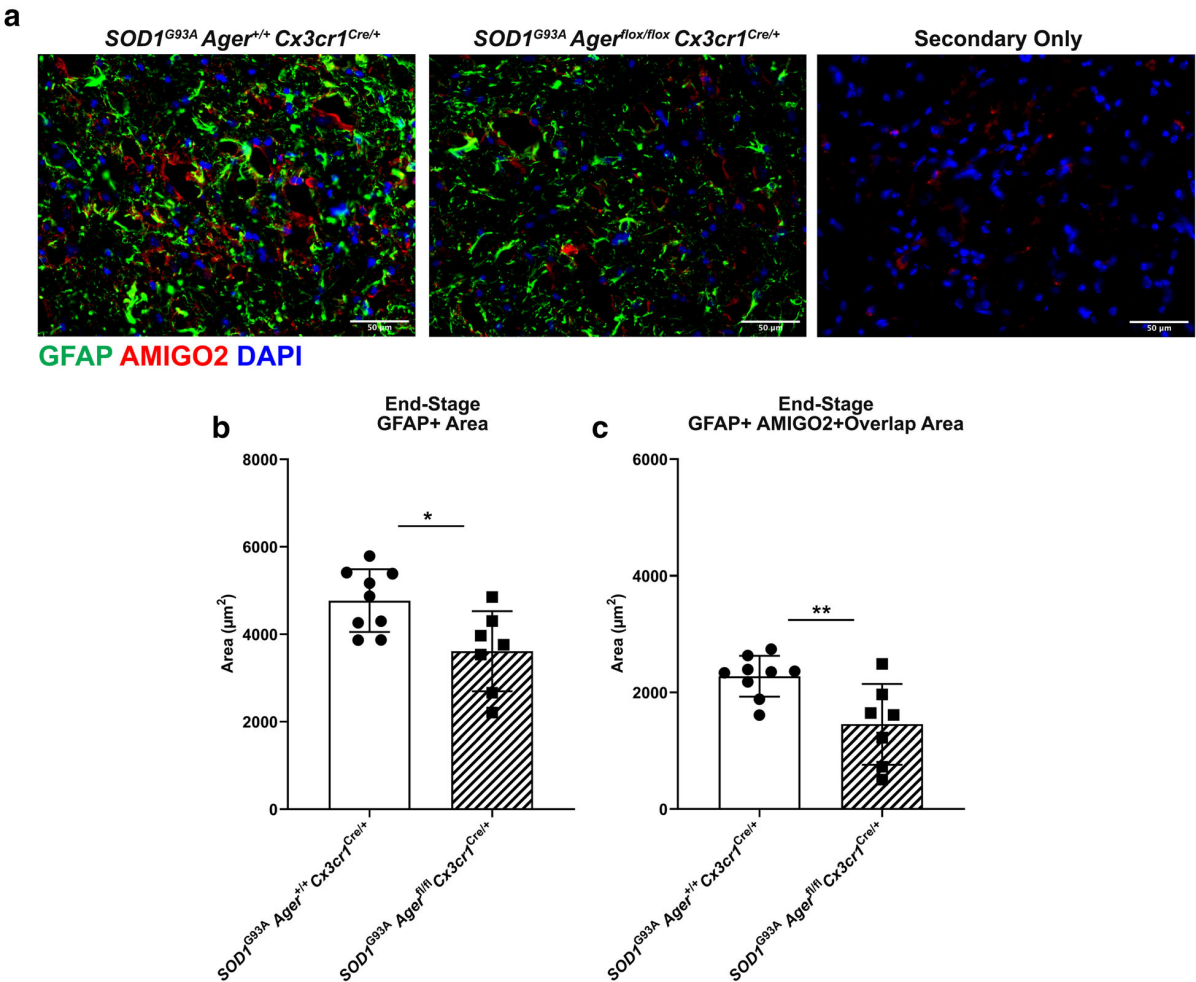
Reactive A1 astrocytes are reduced in male *SOD1*^*G93A*^ mice by microglia *Ager* deletion. **a** Representative images of GFAP and AMIGO2 staining in the lumbar spinal cord ventral horn of the indicated mouse groups. **b** Quantification of GFAP^+^ area. **c** Quantification of GFAP^+^ and AMIGO2^+^ overlap area. Mean *±* SD. *N* = 7 *SOD1*^*G93A*^ *Ager*^fl/fl^ *Cx3cr1*^Cre/+^ mice, and *N* = 9 *SOD1*^*G93A*^ *Ager*^+/+^ *Cx3cr1*^Cre/+^ mice. Independent two sample two-sided *t* test. In **b**, *p = 0.0132. In **c**, **p = 0.0076

The RNA-seq results indicated reduction of a putative causal network involving IL1 (Table 3). In this context, accumulating evidence suggests that microglia-secreted molecules complement component 1q (C1q), IL1α, and TNF can induce astrocyte reactivity and promote neurotoxicity [70, 71]. Prompted by this consideration, we thus investigated if the *SOD1*^*G93A*^ *Ager*^fl/fl^ *Cx3cr1*^Cre/+^ mice displayed alterations in astrocytes. At end-stage, we found that GFAP^+^ area was significantly lower in *SOD1*^*G93A*^ *Ager*^fl/fl^ *Cx3cr1*^Cre/+^ mice relative to Cre-expressing controls (Fig. 9A, B). Recent work has suggested distinct reactive astrocyte phenotypes and identified markers of those states [70, 80]. AMIGO2, adhesion molecule with Ig-like domain 2, was proposed as a marker of “A1” reactive inflammatory astrocytes induced specifically by C1q, IL1α and TNF [70]. Although it is acknowledged that this classification likely does not illuminate the breadth of astrocyte properties and contributions to ALS operative in vivo, we nevertheless examined if the GFAP alterations were concomitant alongside alterations in “A1” astrocytes to begin to define if microglia RAGE might impact astrocyte gene expression. Indeed, we found that GFAP^+^ AMIGO2^+^ overlap area was significantly reduced in *SOD1*^*G93A*^ *Ager*^fl/fl^ *Cx3cr1*^Cre/+^ mice vs. the Cre-expressing controls (Fig. 9A, C). Altogether, these data suggest that microglia *Ager* deletion may reduce astrocytic dysfunction likely through altered cytokine expression.

### Microglia *Ager* deletion reduces microgliosis at an earlier stage in male *SOD1*^*G93A*^ mice

**Fig. 10:**
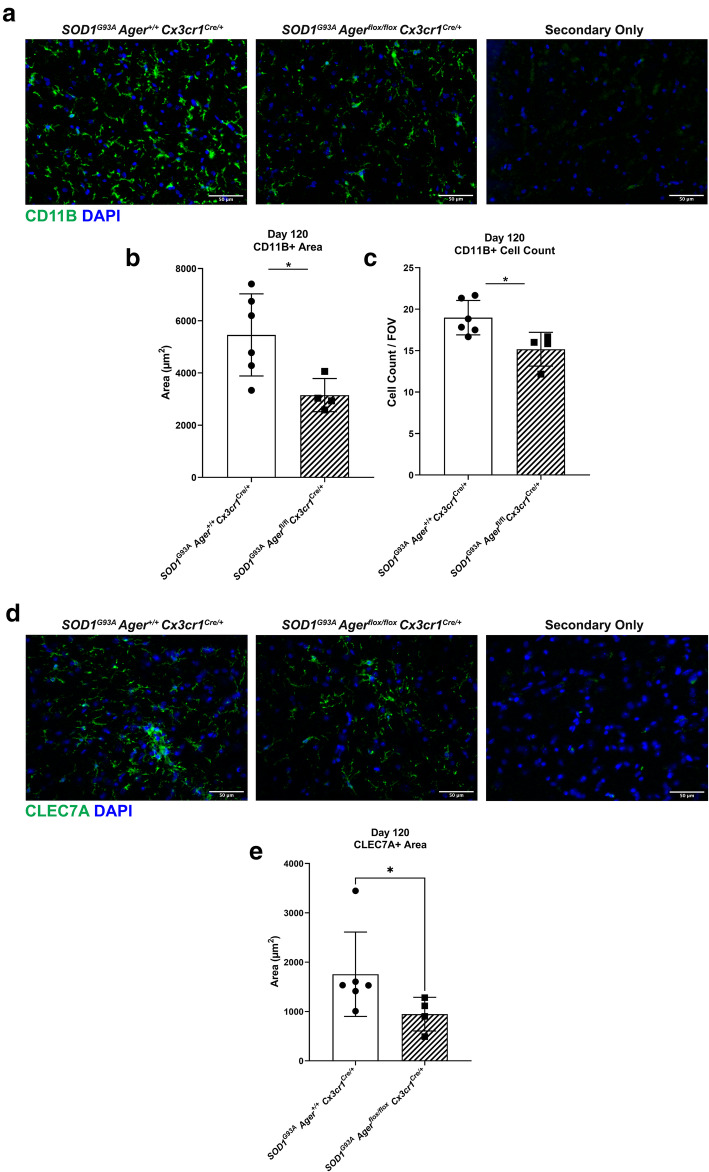
Microglia *Ager *deletion in male *SOD1*^*G93A *^mice reduces microgliosis at 120 days of age. **a** Representative images of CD11B staining in the lumbar spinal cord ventral horn of the indicated mouse groups. **b** Quantification of CD11B^+^ area. **c**. Quantification of CD11B^+^ DAPI^+^ cell number. **d** Representative images of CLEC7A staining in the lumbar spinal cord ventral horn of the indicated mouse groups. **e** Quantification of CLEC7A^+^ area. Mean *±* SD. *N* = 4 *SOD1*^*G93A*^ *Ager*^fl/fl^ *Cx3cr1*^Cre/+^ mice, *N* = 6 *SOD1*^*G93A*^ *Ager*^+/+^ *Cx3cr1*^Cre/+^ mice. In **b**, **c**, independent two sample two-sided *t* test. In **e**, Mann-Whitney *U* test. In **b**, * *p* = 0.0253. In **c**, *p = 0.0212. In **e**, *p = 0.0381

We next considered that it was important to also examine microglia and expression of CLEC7A at a time point within the progression phase, but short of end-stage analysis. It is important to note that microglial CLEC7A expression increases over time in this model, and not all microglia may have notable expression of CLEC7A at 120 days of age [20]. Thus, we addressed this point by examining the expression of both CD11B and CLEC7A at day 120. The results indicated significant reductions in the amount of CD11B^+^ cells and CD11B^+^ area in *SOD1*^*G93A*^ mice devoid of microglia *Ager* (Fig. 10A–C). Similar to the end-stage tissue analysis, we observed reduced CLEC7A^+^ area at day 120 (Fig. 10D, E). Altogether, these data suggested that RAGE expression on microglia may contribute to the promotion of microgliosis (Fig. 10A–D), and may affect CLEC7A expression at a stage within the microglia phenotypic transition but prior to frank end-stage tissue pathologies (Figs. 8A–C and 10D, E). As such, we surmised that the overall RAGE-dependent mechanisms in microglia in *SOD1*^*G93A*^ mice were likely through alterations in microglia cell intrinsic and cell-cell communication pathways between microglia and other cell-types.

Finally, we considered the possibility that the processes affected by microglia *Ager* deletion in *SOD1*^*G93A*^ mice might relate to the processes accompanied by increasing human *AGER* expression in ALS patients. Accordingly, we compared the results of both RNA-seq analyses and found that half of the ingenuity canonical pathways identified in the human data analysis overlapped between the two data sets: “Hepatic fibrosis/Hepatic Stellate Cell Activation,” “Atherosclerosis Signaling,” and “Agranulocyte Adhesion and Diapedesis“ (Tables 1 and 2, Supplemental Figure 6-8). Collectively, although it is acknowledged that the human and mouse spinal cord sequencing experiments were not identically designed, these analyses nevertheless suggest that RAGE may modulate extracellular matrix composition, cell-cell communication and lipid metabolism in ALS spinal cord tissues in patients and in *SOD1*^*G93A*^ mice.

## Discussion

Our study sought to uncover potential RAGE-dependent roles in ALS by utilizing human patient cervical spinal cord RNA-seq data and the *SOD1*^*G93A*^ mouse model of ALS-like pathology. Although RAGE expression was not significantly different between human ALS patients relative to non-neurological controls, this might have been accounted for, in part, by the unexpected observation that ALS patients displayed a range of *AGER* mRNA in the spinal cord. Accordingly, our analysis revealed upregulation of the “AGE-RAGE pathway in diabetic complications” within cervical spinal cord RNA-seq data of ALS vs. control patients. We found that the amount of *AGER* negatively correlated with age at onset and age at death or tracheostomy in ALS patients’ cervical spinal cord. If and to what degree the stratification of ALS patients by spinal cord *AGER* expression may be useful in prediction of prognosis, putative responsiveness to pharmacological interventions and/or the identification of ALS predictive biomarkers, for example, is an important subject for future investigation.

Furthermore, analysis of the consequences of varied degrees of *AGER* expression across patients’ transcriptomic analyses, using *AGER* expression as a continuous variable, indicated enrichment in pathways involved in lipid metabolism, extracellular matrix, and cell-cell communication. In fact, microglia displayed increased RAGE protein overlap within both the anterior white matter and the ventral horn in high *AGER* patients relative to low *AGER* patients. Of note, our data in human ALS did not suggest sex-dependent differences in *AGER-*related gene expression patterns. In parallel, the present work in the murine model indicated that there was a significant increase in overlap of RAGE with microglia in *SOD1*^*G93A*^ mice at the age of 120 days.

Beyond microglia, other cell-types express RAGE in the spinal cord of both *SOD1*^*G93A*^ and human ALS patients. In fact, the roles of RAGE expression in astrocytes are not clear either in homeostasis or in neurodegeneration. RAGE expression in motor neurons has been posited to play a role in the cell-death pathways induced by conditioned media from *SOD1*^*G93A*^*-*expressing astrocytes [81, 82]. A recent report indicated that global constitutive *Ager* deletion caused a decrease in survival of *SOD1*^*G93A*^ mice, while administering a CNS-permeable RAGE antagonist, FPS-ZM1, starting at 60 days of age, reduced gliosis, and increased motor neuron number at 130 days; but did not alter survival in either sex [82]. However, the authors reported a potential sex-dependent impact on the progression of weight loss and time to death following 10% weight loss [82]. In a separate report, another global constitutive *Ager* deleted *SOD1*^*G93A*^ mouse line exhibited increased survival; however, this *Ager* deleted mouse line has been reported to harbor a large genomic duplication, which may have confounded the results and explained the discrepancy between the two studies [83, 84]. However, both studies suggested RAGE inhibition reduced gliosis and improved motor function. Treatment of *SOD1*^*G93A*^ mice with soluble RAGE (sRAGE), which sequesters RAGE ligands, reduced motor pathology, and extended lifespan in *SOD1*^*G93A*^ mice [41]. As there was no significant blood-brain permeability of sRAGE when administered peripherally [85], the mechanism by which sRAGE exerted protective effects remains elusive. It is possible that circulating soluble factors, modulated by sRAGE treatment, entered the CNS and exerted beneficial effects. Altogether, these studies suggest RAGE could exert complex protective and deleterious effects in a time- and location-dependent manner but cell-types responsible for any of these effects are unclear and warrant further study.

Our findings revealed that deletion of microglia *Ager* improved survival and motor function in male but not female *SOD1*^*G93A*^ mice. In this context, in the mouse model employed in our study, the “knock-in” of the Cre recombinase into the *Cx3cr1* locus results in an obligate heterozygous deletion of this gene in all mice, that is fully independent of tamoxifen administration. Thus, all Cre-expressing mice in our study are susceptible to the effects of hemizygous deletion of *Cx3cr1*. After the initiation of the present work, recent studies have independently implicated *Cx3cr1* in the pathogenesis of several neurodegenerative diseases, including ALS. For example, heterozygous deletion of *Cx3cr1* induces changes within microglia reminiscent of aged cells [86–88]. In fact, in a model of Alzheimer disease (AD), heterozygosity of *Cx3cr1* in male mice resulted in reductions of pathology [87]. In contrast, homozygous global deletion of *Cx3cr1* in male but not female *SOD1*^*G93A*^ mice reduced survival [86]. Altogether, these considerations led us to conclude that it was critical to directly compare our findings in microglia *Ager*-deleted mice to the *Cx3cr1-*Cre-control mice to account for the obligate allelic loss of *Cx3cr1*. As we only observed differences in the male mice, we focused further investigation into these groups to determine what may drive the observed phenotypic differences.

Skeletal muscle macrophage content was not significantly different between the genotypes suggesting the beneficial effects of microglia *Ager* deletion were likely restricted to the nervous system. Accordingly, transcriptomic analysis of male *SOD1*^*G93A*^ *Ager*^fl/fl^ *Cx3cr1*^Cre/+^ and Cre-control lumbar spinal cord tissues indicated microglia *Ager* deletion reduced gene expression in pathways related to lipid metabolism, actin cytoskeleton, extracellular matrix, and cell-cell communication. Predicted cytokine regulators of these effects included cytokines, such IL1α, C-X-C motif chemokine ligand 1 (CXCL1), C-X-C motif chemokine ligand 9 (CXCL9), TNF superfamily member 10 (TNFSF10), and IFNs. Several of these cytokines are known to be dysregulated in the context of ALS in murine models and in patients [75–77]. IFN was recently linked to the acquisition of the damage/disease-associated (DAM) microglia phenotype [89]. DAM are known to accumulate in the *SOD1*^*G93A*^ mouse model and are thought to both provoke and ameliorate disease-related pathways [20, 21, 23]. The transition of microglia to this dysfunctional state is theorized to occur in at least several stages with later stages being TREM2-dependent [21]. Critically, CLEC7A, a marker of DAM highly upregulated over time by *SOD1*^*G93A*^ microglia [23], was significantly downregulated via microglia *Ager* deletion at day 120 and in end-stage tissues. Furthermore, the number of CLEC7A^+^ microglia reduced by microglia *Ager* deletion suggested that microglia *Ager* deletion reduces the activation/acquisition of the DAM phenotype or, alternatively, shifts microglia to a novel phenotype. In fact, BV2 microglia-like cells treated with CML-AGE, a canonical RAGE ligand present in the ALS spinal cord, demonstrated a RAGE-dependent increase in *Il1a* and the DEG identified by RNA-seq *Malat1*. *Malat1* has been linked to the regulation of pro-inflammatory responses of peripheral macrophages. *Malat1*-deficient macrophages display reduced LPS responsiveness and altered fibrotic phenotypes [74]. Altogether, these considerations suggest that microglia RAGE may amplify this dysfunctional state, at least in male mice.

Recent work has implicated microglia-sourced IL1α, TNF, and C1q in inducing astrocyte dysfunction and promoting their neurotoxicity in neurodegeneration [70, 90]. In this context, and the considerations that we observe less DAM and that IL1α activity was predicted to be downregulated by microglia *Ager* deletion, we examined the impact of microglia *Ager* deletion on the accumulation of GFAP+ “pan” reactive astrocytes as well as the overlap of GFAP with an “A1” reactive astrocyte marker, AMIGO2 [70, 80]. Consistent with the predicted downregulation of IL1α activity in the transcriptomic data and reductions in DAM, we observed lower GFAP^+^ area and lower overlap area of GFAP^+^ and AMIGO2^+^. These data indicate that microglia RAGE signaling may contribute to transformation of astrocytes to a more neurotoxic phenotype.

As we had performed bulk RNA-seq on the murine lumbar spinal cords at sacrifice, we compared the results between the analysis of the ALS patients and the murine dataset. This exercise revealed that *half* of the ingenuity canonical pathways overlapped between the two analyses and suggests that microglia RAGE may modulate extracellular matrix composition, cell-cell communication, and fatty acid metabolism in ALS and that this may influence disease pathology.

It is important to note that we are unable to distinguish if microglia *Ager* deletion interacted with potential *Cx3cr1-*mediated sex-dependent impacts on pathology, or if microglia RAGE has sex-dependent roles in microglia. In support of the latter, male microglia exhibited an enrichment of the RAGE receptor binding Gene Ontology term relative to female microglia isolated from cortical tissues [91]. Furthermore, recent work indicated sex-dependent actions of HMGB1, a known RAGE ligand, on spinal cord microglia in a model of hypersensitivity [92]. Additionally, recent work indicated unexplained sex-dependent effects of FPS-ZM1 treatment on several measures of progression in *SOD1*^*G93A*^ mice [82]. Yet, the transcriptomic signature we observed in human ALS patients related to *AGER* expression was independent of sex. However, the human patient data analyzed were obtained almost entirely from sporadic ALS patients with no known family history of illness; thus, the generalizability of our sex-dependent findings in this model of familial ALS is unclear. Finally, it is also possible that the observed sex-dependent effect in mice is a result of a complex interaction between roles of RAGE within microglia that were altered due to the developmental loss of one allele of *Cx3cr1*. Further study in distinct models of ALS will be required to discern if microglia RAGE has sex-dependent effects in the pathogenesis of ALS.

Hence, although we may not be able to fully disentangle the effects of the *Cx3cr1* heterozygosity on survival from potential roles for RAGE in pathology the *SOD1*^*G93A*^ mouse model, the direct comparison to the Cre-control reflects the most rigorous design for this work. Importantly, it is known that monocytes also express *Cx3cr1*; however, these cells replenish from *Cx3cr1* deficient pre-cursors within a few days, and monocyte recruitment into the *SOD1*^*G93A*^ spinal cord has been a point of contention within the field. Recent tracing studies have suggested that recruitment is limited until late states of pathology [93]. While we cannot entirely rule out early incipient roles for monocytic RAGE in the context of *SOD1*^*G93A*^ pathology, we predict that the largest benefits were from microglia *Ager* reduction within the CNS. Hence, future studies require testing the RAGE hypothesis in distinct models of ALS, and in a model employing Cre-recombinase mice in which expression of native microglia genes is not affected, and is more specific to microglia, such as the recently developed *Tmem119*-2A-CreERT2 mouse model [94].

Altogether, these data demonstrate that microglia RAGE expression may contribute to microglia dysfunction and ultimately to the disruption of communication with multiple cell types including astrocytes and neurons in the diseased ALS spinal cord. Our findings that motor neuron numbers were higher in lumbar spinal cords of male mice devoid of microglia *Ager* vs. controls at day 120 indicate that such communications emitted from microglia cues were, ultimately, aligned with neuronal survival in the pre-morbid/end-stage state. Collectively, our findings implicate microglia and RAGE in human ALS and provide mechanistic evidence that microglia RAGE contributes to pathology in male *SOD1*^*G93A*^ mice.

## Conclusions

In summary, our findings suggest microglia RAGE may impact human ALS and contribute to ALS-like pathology in male *SOD1*^*G93A*^ mice. Male *SOD1*^*G93A*^ mice bearing microglia *Ager* deficiency from the age of 3 months exhibited reduced gliosis, neuronal and motor function loss, and reduced dysfunctional transcriptomic signatures in lumbar spinal cord tissue. Critically, the observation of a spectrum of *AGER* and RAGE expression in human ALS spinal cord unveils a myriad of areas for research with respect to implications for ALS vulnerability, severity of disease, and, perhaps, responsiveness to RAGE-directed therapeutics. In this very context, the observed overlap between the human and murine dataset analyses indicate that the pathways altered in mice may be relevant in human patient pathologies. In conclusion, these data provide novel evidence that microglia-specific RAGE expression is a disease-modifying factor in ALS, potentially through modulation of the molecular switch from homeostatic microglia to dysfunctional microglia.

## Supplementary Information

**Additional file 1.** Figures and figure legend for Supplemental Figure 1–8. Also, table legends for Supplemental Tables 1.1–1.10.

**Additional file 2.** Excel workbook containing Supplemental Tables 1.1–1.10.

## Data Availability

Murine raw FastQ sequencing data and corresponding normalized count data are uploaded for public access on the NCBI GEO database under accession number: GSE160402. All raw Human RNA-seq data from Target ALS samples are publicly available via The Target ALS Multicentered Postmortem Tissue Core, the New York Genome Center for Genomics of Neurodegenerative Disease, Amyotrophic Lateral Sclerosis Association and TOW Foundation (www.targetals.org). Access can be requested by emailing ALSData@nyugenome.org.

## Abbreviations

AGE: Advanced glycation end product
ALS: Amyotrophic lateral sclerosis
AMIGO2: Adhesion molecule with Ig like domain 2
C1q: Complement component 1q
CD11B: Integrin subunit alpha m
ChAT: Choline acetyltransferase
CLEC7A: c-Type lectin domain containing 7a protein (or dectin-1)
CML: Carboxymethyllysine
CNS: Central nervous system
CXCL1: C-X-C motif chemokine ligand 1
CXCL9: C-X-C motif chemokine ligand 9
DAM: Damage/disease-associated microglia
DIAPH1: Diaphanous related formin 1
fALS: Familial amyotrophic lateral sclerosis
GFAP: Glial fibrillary acidic protein
HMGB1: High-mobility group box 1
hSOD1: Human SOD1
IBA1: Ionized calcium-binding adapter molecule 1
IFN: Interferon
IHC: Immunohistochemistry
IL1: Interleukin 1
IPA: Ingenuity pathway analysis
MAP 2: Microtubule-associated protein 2
NeuN: Neuronal nuclei antigen
RAGE: Receptor for advanced glycation end products
sALS: Sporadic amyotrophic lateral sclerosis
SOD1: Superoxide dismutase 1
sRAGE: Soluble RAGE
TAM: Tamoxifen
TREM2: Triggering receptor expressed on myeloid cells 2
WT: Wild-type
