# Supplementary material for: Microglia RAGE exacerbates the progression of neurodegeneration within the SOD1G93A murine model of amyotrophic lateral sclerosis in a sex-dependent manner
Source: J Neuroinflammation. 2021 Jun 15;18:139. doi: 10.1186/s12974-021-02191-2 (PMC8207569; doi:10.1186/s12974-021-02191-2)
Supplement: Supplementary file 1 — Additional file 1. Figures and figure legend for Supplemental Figure 1–8. Also, table legends for Supplemental Tables 1.1–1.10. [file 12974_2021_2191_MOESM1_ESM.zip › Additional file 1/AdditionalFile1_revised_FINAL052721.docx]

***Supplemental Data***

*Supplemental Figure 1: Microglia Ager deleted SOD1^G93A^ mice exhibit no change in overlap between RAGE and GFAP or MAP2.*

**A.** Representative images of GFAP and RAGE staining in the ventral horn of *SOD1^G93A^* mouse lumbar spinal cord at end-stage. **B.** Quantification of GFAP and RAGE overlap area at end-stage. **C.** Representative images of MAP2 and RAGE staining in the ventral horn of *SOD1^G93A^* mouse lumbar spinal cord at day120. **D.** Quantification of MAP2 and RAGE overlap area at day 120. In **A-B**, *N*=6 *SOD1^G93A^* *Ager*^fl/fl^ *Cx3cr1*^Cre/+^ mice, *N*=8 *SOD1^G93A^* *Ager*^+/+^ *Cx3cr1*^Cre/+^ mice. In **C-D**, *N*=4 *SOD1^G93A^* *Ager*^fl/fl^ *Cx3cr1*^Cre/+^ mice, *N*=6 *SOD1^G93A^* *Ager*^+/+^ *Cx3cr1*^Cre/+^ mice. Scale bar: 50 µm.

*Supplemental Figure 2: Microglia Ager deleted SOD1^G93A^ mice exhibit no change in onset.*

**A.** Kaplan–Meier estimates of percent of male mice affected by onset of pathology plotted vs. age (days). *N*=16 *SOD1^G93A^* *Ager*^fl/fl^ *Cx3cr1*^Cre/+^ mice, and *N*=13 *SOD1^G93A^ Ager*^+/+^ *Cx3cr1*^Cre/+^ mice. **B.** Kaplan–Meier estimates of percent of female mice affected by onset of pathology plotted vs. age (days). *N*=15 *SOD1^G93A^* *Ager*^fl/fl^ *Cx3cr1*^Cre/+^ mice and *N*=13 *SOD1^G93A^ Ager*^+/+^ *Cx3cr1*^Cre/+^ mice. The Logrank test was performed to compare the survival distributions between groups.

*Supplemental Figure 3: Microglia Ager deleted SOD1^G93A^ mice exhibit no changes in skeletal muscle macrophages in end-stage gastrocnemius muscle.*

**A.** Representative images of F4/80 staining in *SOD1^G93A^* murine gastrocnemius muscle at end-stage. Scale bar: 50 µm. **B.** Quantification of F4/80 area. **C.** Representative images of CD68 staining in *SOD1^G93A^* murine gastrocnemius muscle at end-stage. Scale bar: 50 µm. **D.** Quantification of CD68 area. *N*=7 *SOD1^G93A^* *Ager*^fl/fl^ *Cx3cr1*^Cre/+^ mice, *N*=5 *SOD1^G93A^* *Ager*^+/+^ *Cx3cr1*^Cre/+^ mice.

*Supplemental Figure 4: Microglia Ager deleted SOD1^G93A^ mice exhibit no changes in skeletal muscle macrophages in day 120 gastrocnemius muscle.*

**A.** Representative images of F4/80 staining in *SOD1^G93A^* murine gastrocnemius muscle at day 120. Scale bar: 50 µm. **B.** Quantification of F4/80 area. **C.** Representative images of CD68 staining in *SOD1^G93A^* murine gastrocnemius muscle at day 120. Scale bar: 50 µm. **D.** Quantification of CD68 area. *N*=4 *SOD1^G93A^* *Ager*^fl/fl^ *Cx3cr1*^Cre/+^ mice, *N*=6 *SOD1^G93A^* *Ager*^+/+^ *Cx3cr1*^Cre/+^ mice.

*Supplemental Figure 5: BV2 cells up-regulate Il1a and Malat1 expression in a RAGE-dependent manner.*

**A.** Heatmap illustrating fold change in relative fluorescence units (RFU) of specified cytokines relative to *18s rRNA* across the indicated groups. *N*=3 biological replicates pooled per group. **B.** Quantification of *Il1a* expression across the indicated groups relative to HSA-DMSO. *N=3* independent biological replicates/group. **C.** Quantification of *Ager* expression in lentiviral transduced BV2 cells relative to Scramble expressing controls. *N=3* Scramble, *N=4* sh*Ager*. Independent two sample two-sided *t*-test. **D.** Quantification of *Il1a* expression across the indicated groups relative to HSA-DMSO. *N=4* HSA-sh*Ager*, CML-sh*Ager*, CML-Scramble, and *N*=3 HSA-Scramble. **E.** Quantification of *Malat1* expression across the indicated groups relative to HSA-DMSO. *N=4/group.* In **B**,**D-E***,* One-way ANOVA with post-hoc Holm-Šídák multiple comparisons test. In **B**, *****p<0.0001, *p=0.0146.* In **C**, **p=0.0198.* In **D**, **p=*0.0499 and **p*=0.0395 from left to right. In **E,** **p=*0.0426 and **p*=0.0242 from left to right.

*Supplemental Figure 6: Visualization of changes to Atherosclerosis Signaling pathway.*

Atherosclerosis Signaling IPA pathway. Colored by log_2_ fold change. *SOD1^G93A^ Ager*^+/+^ *Cx3cr1*^Cre/+^ vs. *SOD1^G93A^* *Ager*^fl/fl^ *Cx3cr1*^Cre/+^ mice**.** *N*=4 mice/group.

*Supplemental Figure 7: Visualization of changes to Hepatic fibrosis / Hepatic Stellate Cell Activation pathway.*

Hepatic fibrosis / Hepatic Stellate Cell Activation IPA pathway. Colored by log_2_ fold change. *SOD1^G93A^ Ager*^+/+^ *Cx3cr1*^Cre/+^ vs. *SOD1^G93A^* *Ager*^fl/fl^ *Cx3cr1*^Cre/+^ mice**.** *N*=4 mice/group.

*Supplemental Figure 8: Visualization of changes to Agranulocyte Adhesion and Diapedesis pathway.*

Agranulocyte Adhesion and Diapedesis IPA pathway. Colored by log_2_ fold change. *SOD1^G93A^ Ager*^+/+^ *Cx3cr1*^Cre/+^ vs. *SOD1^G93A^* *Ager*^fl/fl^ *Cx3cr1*^Cre/+^ mice**.** *N*=4 mice/group.

*Supplemental Tables (Additional File 2).*

**Table.1.1** Differentially expressed genes between ALS and Control Patients.

Differential expression results comparing ALS MND and Control patients (*N*=76 and 11) controlling for sex of the patients in the model. Genes with FDR<0.05 are listed. Positive values indicate up-regulation in ALS patients.

**Table1.2** CAMERA analysis between ALS and Control Patients.

Result from CAMERA analysis for all KEGG pathways and GO terms of ALS and Control patients (*N*=76 and 11) controlling for sex of the patients in the model.

**Table1.3** ROAST analysis between ALS and Control Patients.

Result from ROAST analysis for all KEGG pathways and GO terms of ALS and Control patients (*N*=76 and 11) controlling for sex of the patients in the model.

**Table1.4** Available Metadata from ALS Patients.

**Table1.5** Differentially expressed genes in ALS patients altered with changing *AGER* expression.

Differential expression utilizing *AGER* as a continuous predictor in ALS patients (*N*=76). Positive values indicate up-regulation with increasing *AGER* expression.

**Table1.6** Overrepresentation analysis dependent on *AGER* expression in ALS patients.

Overrepresentation results of KEGG pathways dependent on utilizing *AGER* as a continuous predictor. Input genes were significantly differentially-expressed genes with FDR<0.05 and |Log_2_ fold change| ≥ 0.5 . Positive values indicate up-regulation in with increasing *AGER* expression.

**Table1.7** Differentially expressed genes between microglia *Ager* deficient and Cre-expressing control male *SOD1^G93A^* mice.

Differential expression results comparing *SOD1^G93A^ Ager*^fl/fl^ *Cx3cr1*^Cre/+^ and *SOD1^G93A^ Ager*^+/+^ *Cx3cr1*^Cre/+^ mice (*N*=4/group). Genes with FDR<0.1 are listed.

**Table1.8** Overrepresentation analysis of KEGG pathways between microglia *Ager* deficient and Cre-expressing control male *SOD1^G93A^* mice.

Overrepresentation results of KEGG pathways comparing *SOD1^G93A^ Ager*^fl/fl^ *Cx3cr1*^Cre/+^ and *SOD1^G93A^* *Ager*^+/+^ *Cx3cr1*^Cre/+^ mice (*N*=4/group). Input genes were significantly differentially-expressed genes with FDR<0.05. Positive values indicate up-regulation in *SOD1^G93A^* *Ager*^+/+^ *Cx3cr1*^Cre/+^ mice.

**Table1.9** Overrepresentation analysis of canonical pathways between microglia *Ager* deficient and Cre-expressing control male *SOD1^G93A^* mice.

Overrepresentation results of ingenuity canonical pathways from IPA analyses comparing *SOD1^G93A^* *Ager*^fl/fl^ *Cx3cr1*^Cre/+^ and *SOD1^G93A^ Ager*^+/+^ *Cx3cr1*^Cre/+^ mice (*N*=4/group). Input genes were significantly differentially-expressed genes with FDR<0.05.

**Table1.10** Causal network analysis between microglia *Ager* deficient and Cre-expressing control male *SOD1^G93A^* mice.

Causal network and master regulator prediction from IPA analyses comparing *SOD1^G93A^ Ager*^fl/fl^ *Cx3cr1*^Cre/+^ and *SOD1^G93A^* *Ager*^+/+^ *Cx3cr1*^Cre/+^ mice (*N*=4/group). Input genes were significantly differentially-expressed genes with FDR<0.05.
