# Supplementary figures and images for: Microglia RAGE exacerbates the progression of neurodegeneration within the SOD1G93A murine model of amyotrophic lateral sclerosis in a sex-dependent manner
Source: J Neuroinflammation. 2021 Jun 15;18:139. doi: 10.1186/s12974-021-02191-2 (PMC8207569; doi:10.1186/s12974-021-02191-2)

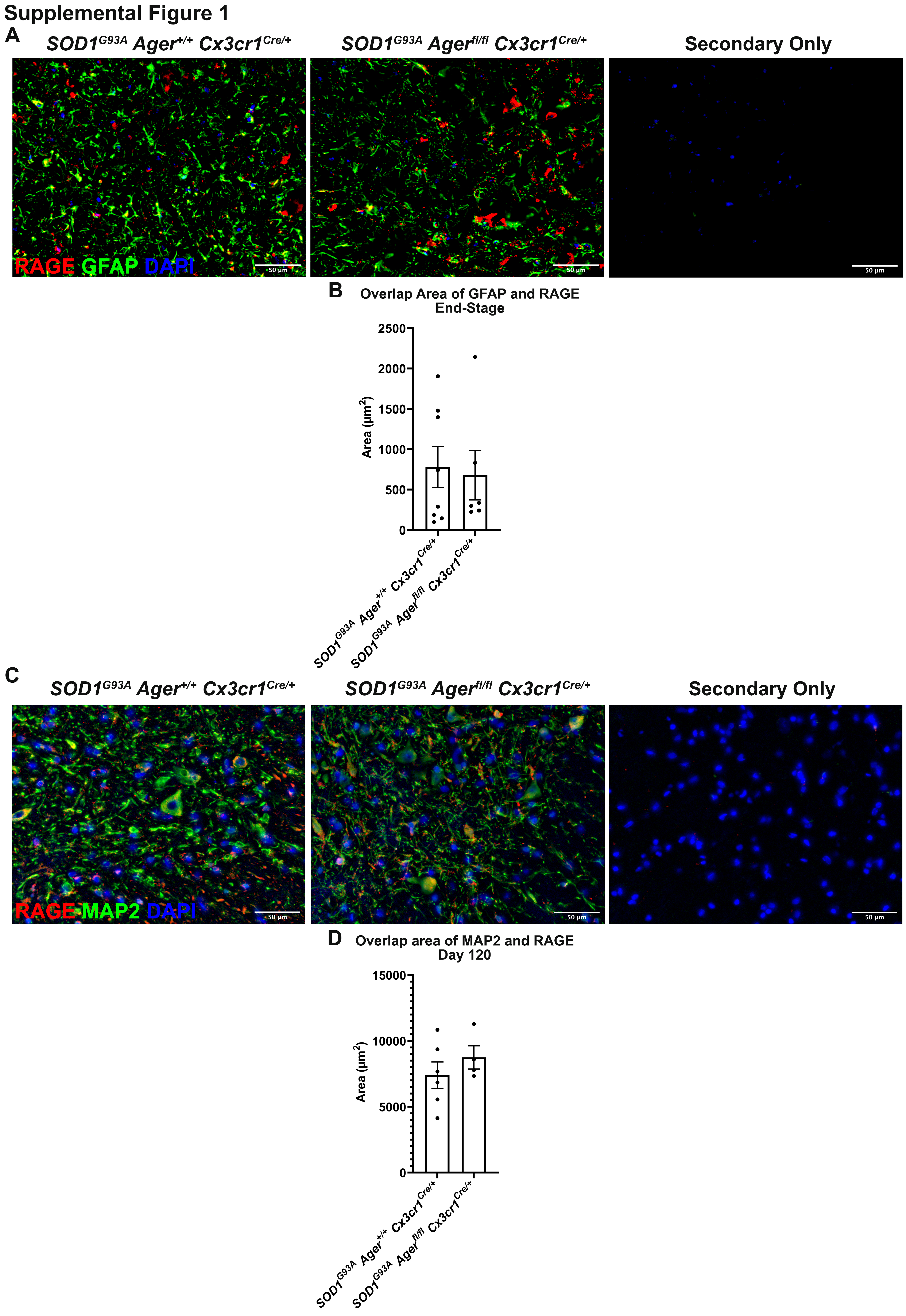

Supplement: Supplementary file 1 — Additional file 1. Figures and figure legend for Supplemental Figure 1–8. Also, table legends for Supplemental Tables 1.1–1.10. [file 12974_2021_2191_MOESM1_ESM.zip › Additional file 1/Supplemental Figure 1-052621.tif]

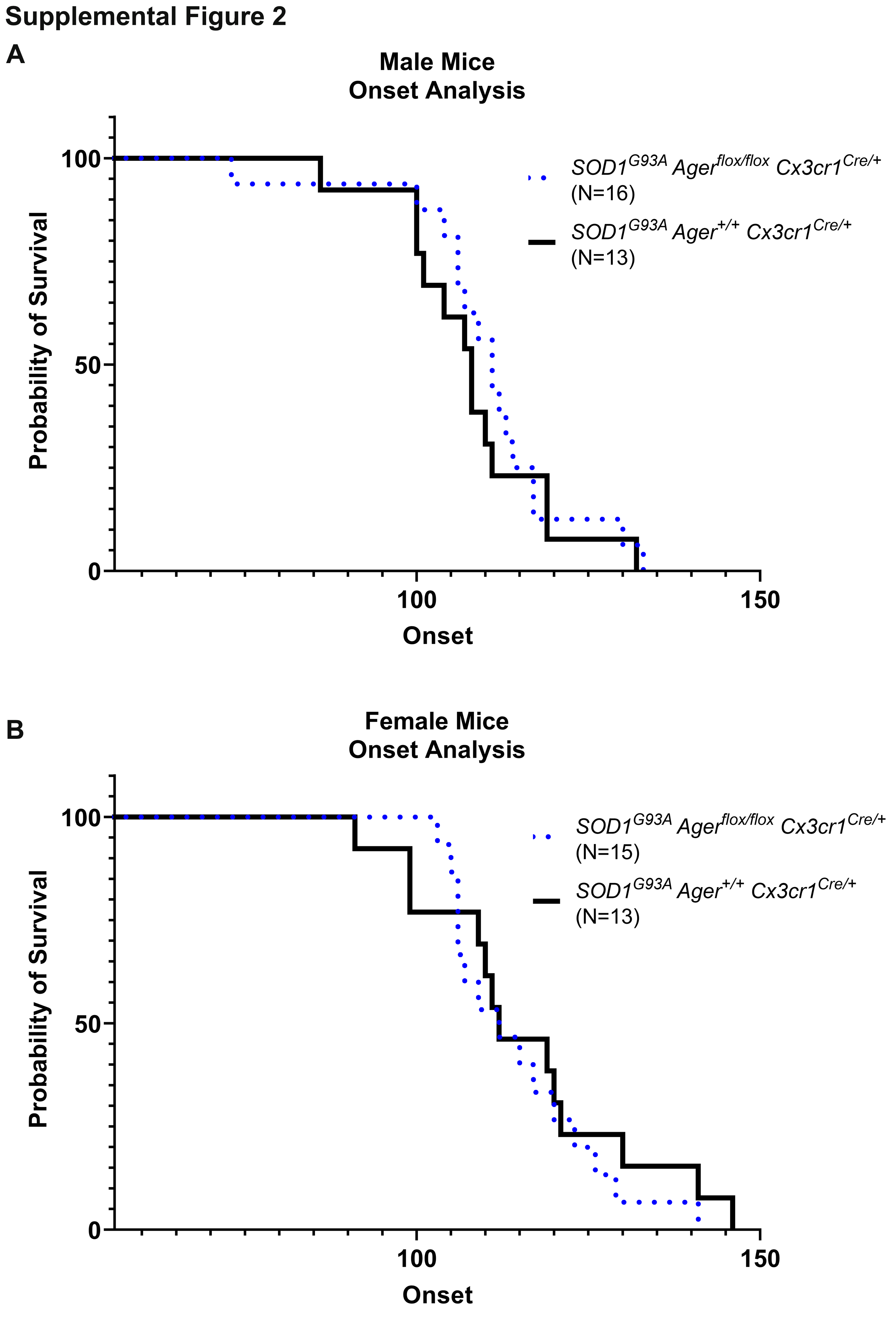

Supplement: Supplementary file 1 — Additional file 1. Figures and figure legend for Supplemental Figure 1–8. Also, table legends for Supplemental Tables 1.1–1.10. [file 12974_2021_2191_MOESM1_ESM.zip › Additional file 1/Supplemental Figure 2-042621.tif]

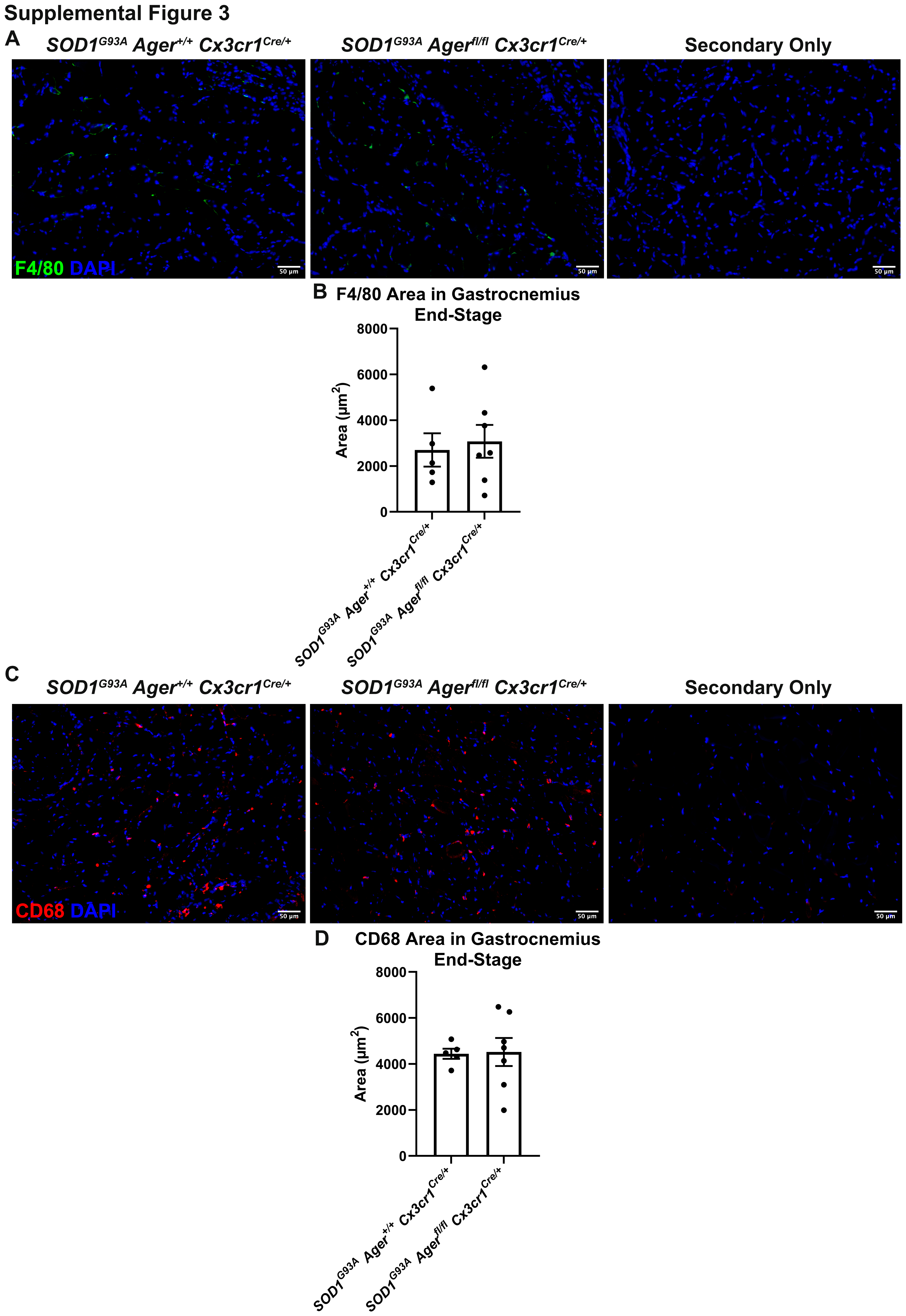

Supplement: Supplementary file 1 — Additional file 1. Figures and figure legend for Supplemental Figure 1–8. Also, table legends for Supplemental Tables 1.1–1.10. [file 12974_2021_2191_MOESM1_ESM.zip › Additional file 1/Supplemental Figure 3-052621.tif]

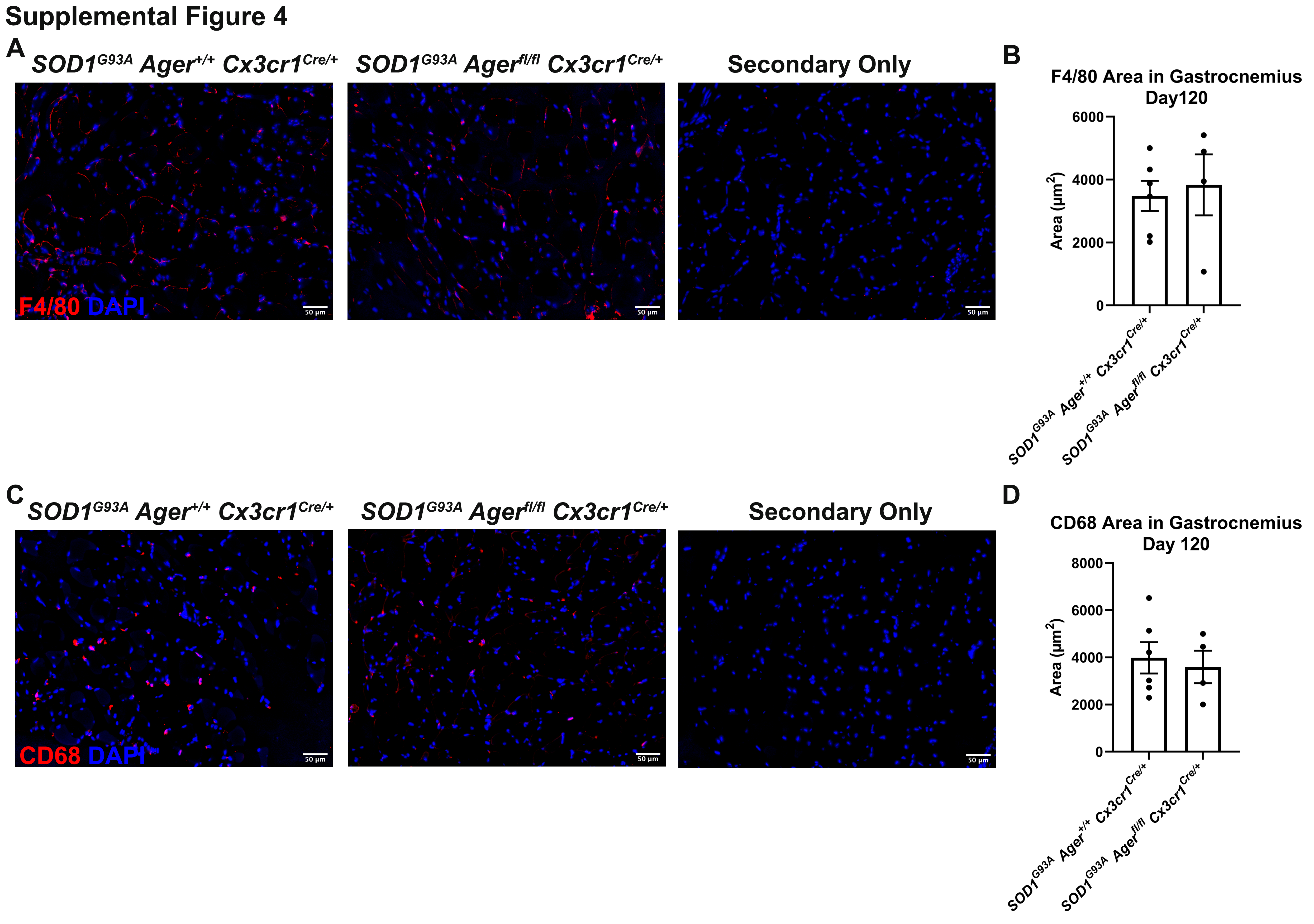

Supplement: Supplementary file 1 — Additional file 1. Figures and figure legend for Supplemental Figure 1–8. Also, table legends for Supplemental Tables 1.1–1.10. [file 12974_2021_2191_MOESM1_ESM.zip › Additional file 1/Supplemental Figure 4-050421.tif]

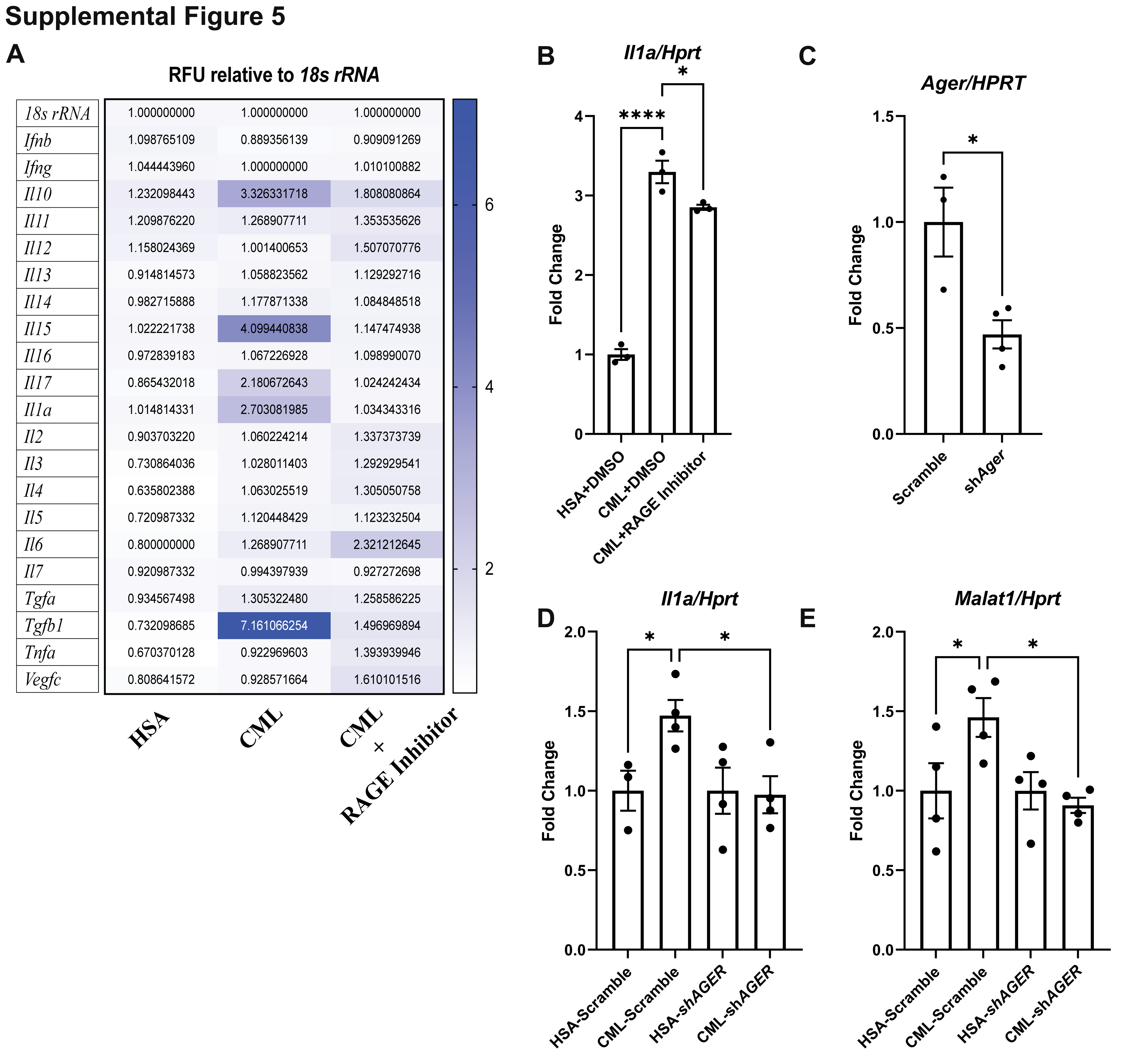

Supplement: Supplementary file 1 — Additional file 1. Figures and figure legend for Supplemental Figure 1–8. Also, table legends for Supplemental Tables 1.1–1.10. [file 12974_2021_2191_MOESM1_ESM.zip › Additional file 1/Supplemental Figure 5-042921.tif]

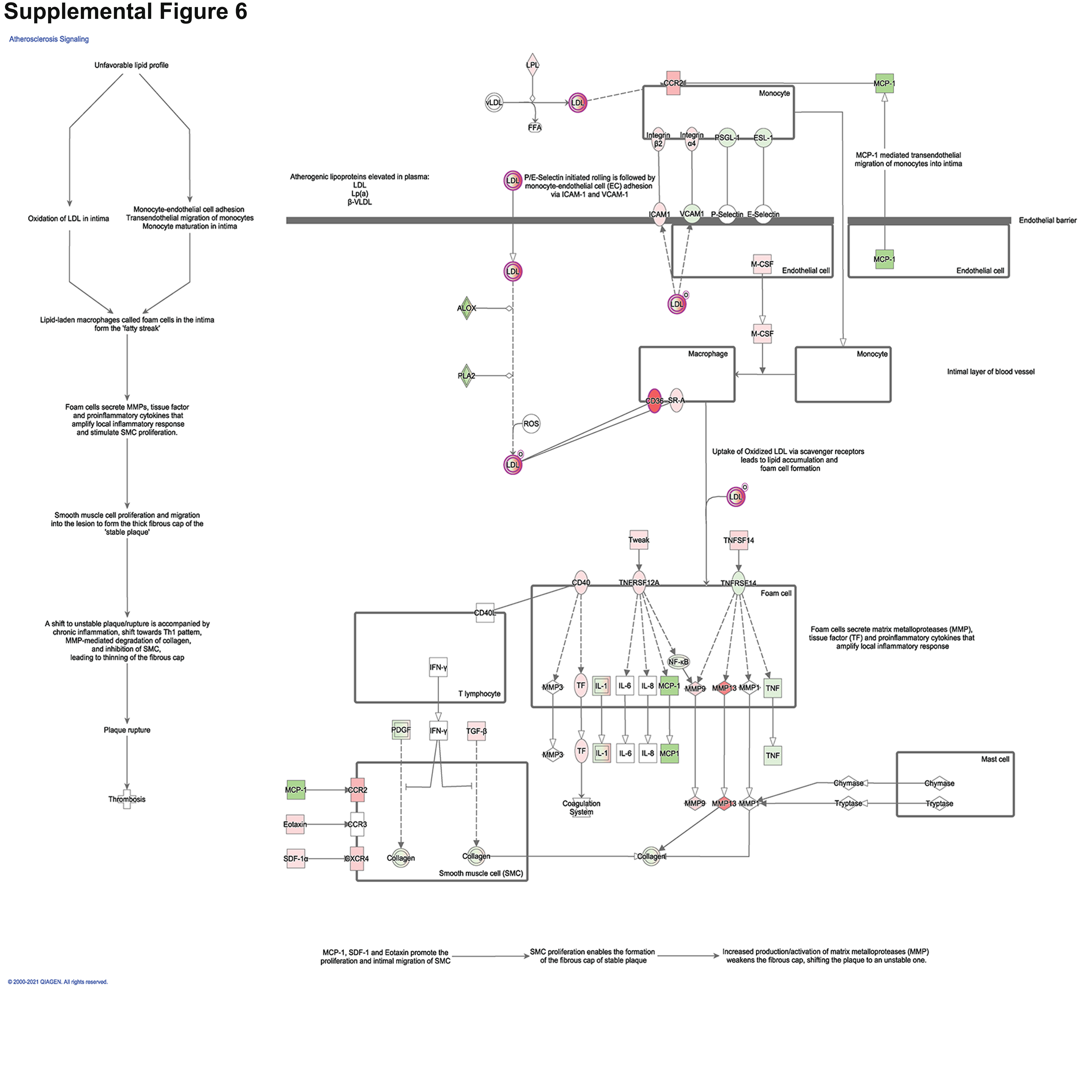

Supplement: Supplementary file 1 — Additional file 1. Figures and figure legend for Supplemental Figure 1–8. Also, table legends for Supplemental Tables 1.1–1.10. [file 12974_2021_2191_MOESM1_ESM.zip › Additional file 1/Supplemental Figure 6-042721.tif]

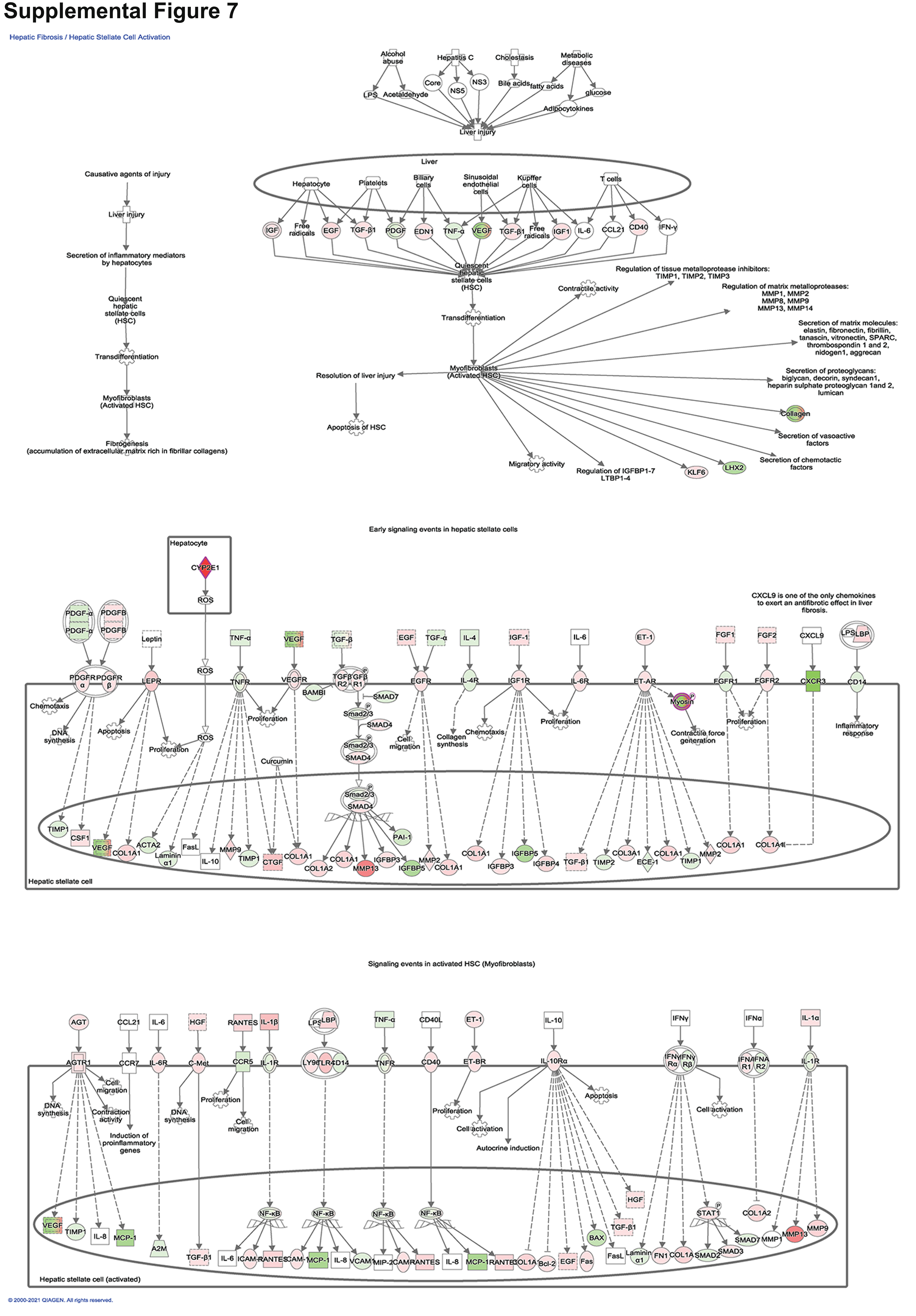

Supplement: Supplementary file 1 — Additional file 1. Figures and figure legend for Supplemental Figure 1–8. Also, table legends for Supplemental Tables 1.1–1.10. [file 12974_2021_2191_MOESM1_ESM.zip › Additional file 1/Supplemental Figure 7-042721.tif]

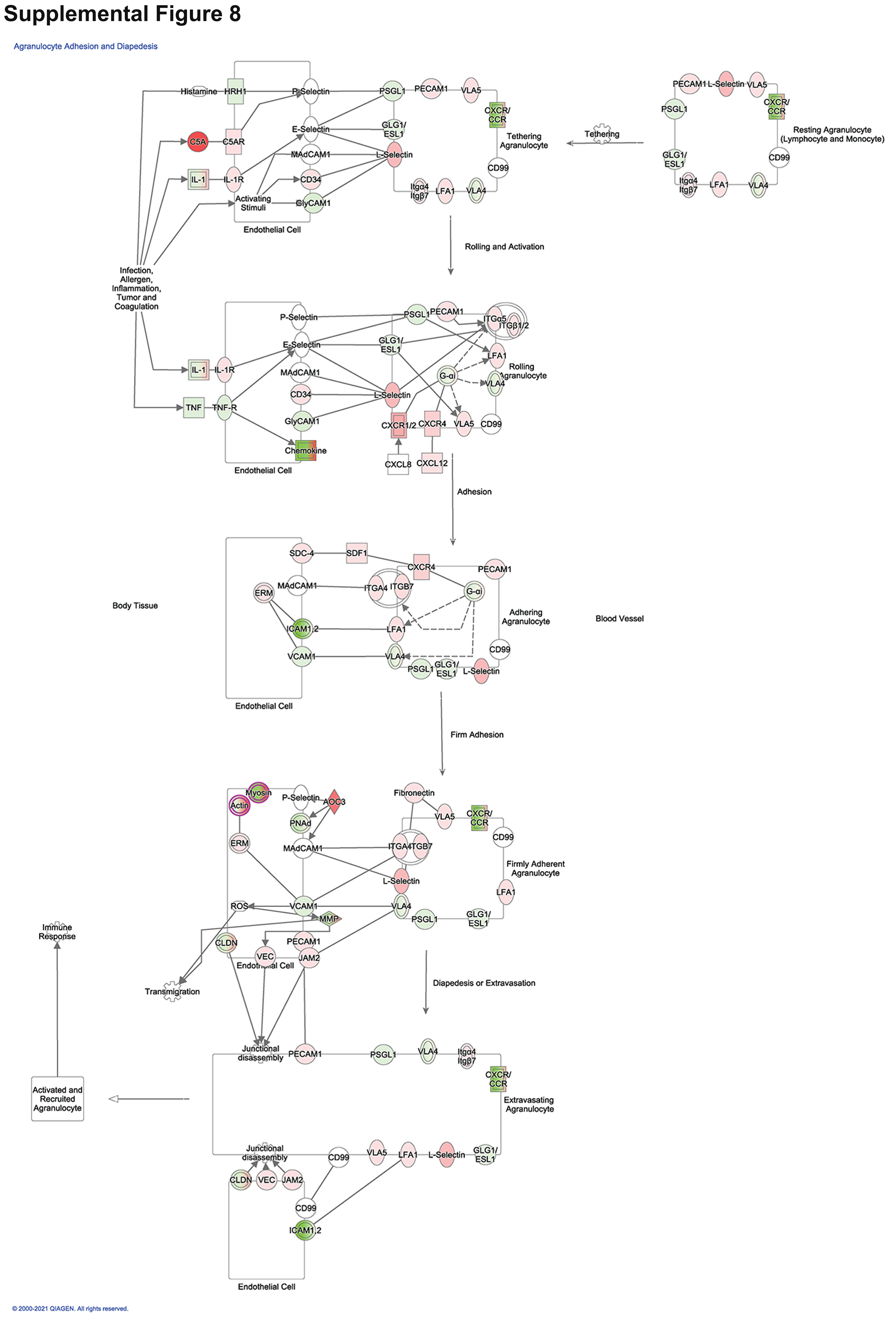

Supplement: Supplementary file 1 — Additional file 1. Figures and figure legend for Supplemental Figure 1–8. Also, table legends for Supplemental Tables 1.1–1.10. [file 12974_2021_2191_MOESM1_ESM.zip › Additional file 1/Supplemental Figure 8-042721.tif]
